# The usage of data in NHS primary care commissioning: a realist review

**DOI:** 10.1186/s12916-023-02949-w

**Published:** 2023-07-03

**Authors:** Alexandra Jager, Geoff Wong, Chrysanthi Papoutsi, Nia Roberts

**Affiliations:** 1 Nuffield Department of Primary Care Health Sciences, University of Oxford, Oxford, UK; 2 Bodleian Health Care Libraries, Medical Sciences, University of Oxford, Oxford, UK

**Keywords:** Commissioning, Realist, Systematic review, Qualitative

## Abstract

**Background:**

Primary care has been described as the ‘bedrock’ of the National Health Service (NHS) accounting for approximately 90% of patient contacts but is facing significant challenges. Against a backdrop of a rapidly ageing population with increasingly complex health challenges, policy-makers have encouraged primary care commissioners to increase the usage of data when making commissioning decisions. Purported benefits include cost savings and improved population health. However, research on evidence-based commissioning has concluded that commissioners work in complex environments and that closer attention should be paid to the interplay of contextual factors and evidence use. The aim of this review was to understand how and why primary care commissioners use data to inform their decision making, what outcomes this leads to, and understand what factors or contexts promote and inhibit their usage of data.

**Methods:**

We developed initial programme theory by identifying barriers and facilitators to using data to inform primary care commissioning based on the findings of an exploratory literature search and discussions with programme implementers. We then located a range of diverse studies by searching seven databases as well as grey literature. Using a realist approach, which has an explanatory rather than a judgemental focus, we identified recurrent patterns of outcomes and their associated contexts and mechanisms related to data usage in primary care commissioning to form context-mechanism-outcome (CMO) configurations. We then developed a revised and refined programme theory.

**Results:**

Ninety-two studies met the inclusion criteria, informing the development of 30 CMOs. Primary care commissioners work in complex and demanding environments, and the usage of data are promoted and inhibited by a wide range of contexts including specific commissioning activities, commissioners’ perceptions and skillsets, their relationships with external providers of data (analysis), and the characteristics of data themselves. Data are used by commissioners not only as a source of evidence but also as a tool for stimulating commissioning improvements and as a warrant for convincing others about decisions commissioners wish to make. Despite being well-intentioned users of data, commissioners face considerable challenges when trying to use them, and have developed a range of strategies to deal with ‘imperfect’ data.

**Conclusions:**

There are still considerable barriers to using data in certain contexts. Understanding and addressing these will be key in light of the government’s ongoing commitments to using data to inform policy-making, as well as increasing integrated commissioning.

**Supplementary Information:**

The online version contains supplementary material available at 10.1186/s12916-023-02949-w.

## Background

Evidence-based policy refers to the different ways in which policy decisions and initiatives can be supported or influenced by (research) evidence [1]. Purported benefits include greater workforce productivity, more efficient use of public resources, and higher likelihood of implementing successful programmes [2, 3]. A wide variety of qualitative and quantitative evidence can be used in evidence-based policy, and the perceived utility of evidence can vary by stakeholder [2].

Within the English National Health Service (NHS), those responsible for delivering evidence-based policy include healthcare commissioners [4]. Commissioning refers to the proactive and strategic process of planning, purchasing, contracting, and monitoring of health services to meet population health needs [5, 6]. The term ‘primary care commissioning’ can refer to both commissioning led by primary care as well as the commissioning of primary care services themselves (which is the focus of this review), i.e. the commissioning of services provided within general practice [7] and of other primary medical services, i.e. dentistry, community pharmacy and ophthalmology services [8]. Since 2022, integrated care boards (ICBs) and integrated care systems (ICSs) have been legal entities with statutory powers and responsibilities tasked with delivering joined-up support and care, with the former taking on responsibility for primary care commissioning [7, 9]. With general practice accounting for 90% of patient consultations (while receiving about 8% of the NHS budget), primary care commissioning is integral to the sustainability of the NHS as whole, given that general practitioners prevent overuse of more expensive health services and enable provision of cost-effective treatment [10–13].

Legal requirements and practical support are in place to promote evidence-based policy and commissioning, including the 2012 Health and Social Care Act which created a statutory duty for the usage of research evidence to help improve patient outcomes and achieve value for money [14, 15]. NHS England established Data Services to ensure that information about the performance and impact of NHS services was available to commissioners [16]. In addition, numerous guides and toolkits have been developed to promote and facilitate evidence-based commissioning, such as NHS RightCare and the NHS Atlas of Variation in Healthcare [15, 17–19].

It has been argued that evidence-based commissioning is particularly pertinent as the NHS is facing severe financial and demographic challenges, with costs in expenditure expected to rise in the medium- to long-term even according to conservative estimates [20, 21]. Some health economists have argued that in order to remain financially sustainable, commissioners must utilise data on healthcare expenditure and understand drivers of variation of activity to help achieve significant cost savings [20, 22]. Despite policy commitment to using data as a key enabler of cost savings and improved health outcomes, the limited research literature on this topic has criticised the utility of the data available and found that commissioners encounter challenges using them meaningfully [17, 23, 24]. There is also limited research on the usage of evidence and decision making in British healthcare commissioning [5, 25]. A recurrent theme in existing research is that evidence use in commissioning is a multifaceted and complex process that can vary by person and context, that evidence is not always used to inform decision-making [4, 5, 26], and that attention should be placed on understanding the role of context affecting evidence use [4, 27, 28].

In this review, we focus on data, defined as quantitative information, including nominal data, and seek to unpack the complex and context-dependent processes underpinning the usage (or not) of data in commissioning. We aim to make a novel contribution by asking:

- How and why do commissioners use data to inform primary care commissioning?
- What outcomes does this lead to?
- What factors or contexts promote and inhibit the usage of data in primary care commissioning decisions?

## Methods

### Review process

We used a realist synthesis approach, i.e. a theory-driven approach based on a realist philosophy of science, with particular emphasis on understanding causation [29]. This attempts to unpack the relationships between contexts, mechanisms, and outcomes to understand failures, successes, and other possible intended and unintended outcomes of programmes and interventions [29]. Recurrent patterns of outcomes (or demi-regularities) and associated contexts and mechanisms are, where possible, linked to substantive theories, thus forming context-mechanism-outcome (CMO) configurations [29, 30]. The five review steps are based on Pawson’s suggested iterative steps and those outlined by Papoutsi et al. [31–33]. We provide a brief summary of these steps, with a more detailed overview provided in Additional file 1 [34–50].

This review aligns with the Realist And MEta-narrative Evidence Syntheses: Evolving Standards (RAMESES) publication standards [29].

#### Step 1: Locate existing theories

Initial programme theory (Additional file 2) [4, 6, 17, 26, 51–62] was built by identifying barriers and facilitators to using data to inform primary care commissioning contained in 15 studies located via an exploratory literature search (Additional file 3) and informal conversations with a former and current NHS commissioner and a public health worker.

#### Step 2: Search for evidence

Studies were identified via three broad and interdisciplinary formal literature searches completed between March 2019 and March 2022 (Additional file 3) and reference linking.

#### Step 3: Select studies

The criteria outlined to select studies are shown in Table 1.

**Table 1:** Inclusion and exclusion criteria

Inclusion criteria	Exclusion criteria
The article is written in English	The article is written in a language other than English
The article describes how data have been used, either alone or in conjunction with other forms of evidence, to make, inform, or influence decisions about commissioning primary care services within the NHS in England	The article does not describe how data have been used to make, inform, or influence decisions about commissioning primary care services within the NHS in England
The article describes how data were actually used to make, inform, or influence decisions about commissioning primary care services within the NHS in England	The article describes how data could or should be used to make, inform, or influence decisions about commissioning primary care services within the NHS in England

#### Step 4: Extract and organise data

Following full-text screening, we imported the included studies (Additional file 4) into NVivo 12 [39] (a qualitative data management software package) for coding.

#### Step 5: Synthesise the evidence according to a realist logic of analysis

We initially coded all relevant concepts and ideas as well as any substantive theories mentioned in the included studies. Studies were then re-read, with several forms of reasoning were used to identify contexts, mechanisms, and outcomes, namely induction, deduction, retroduction, and abduction. Once draft CMOs had been developed, we used several forms of reasoning as suggested by Pawson, namely juxtaposition, reconciliation, consolidation, and situating, to further refine and develop the CMOs (see Additional file 1 for further details).

## Results

**Fig. 1:**
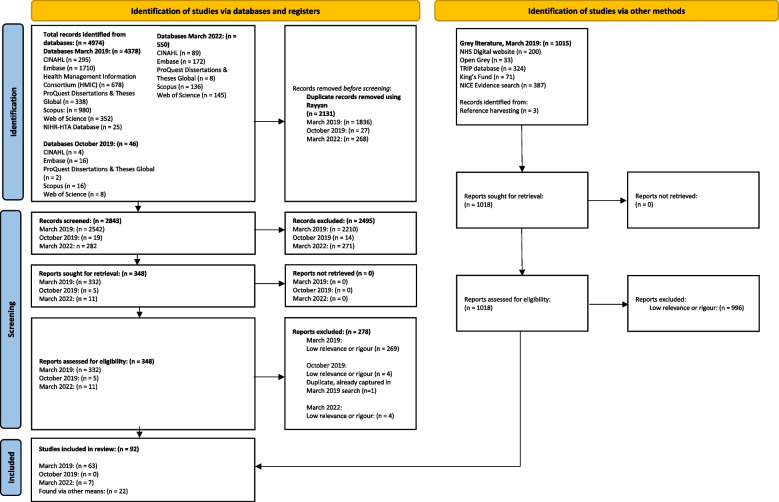
PRISMA flow chart

Following de-duplication, the searches identified 3852 studies for screening. Ninety-two studies were included in the review (Fig. 1). As outlined in Table 2, most of the studies either used qualitative or mixed methods approaches.

**Table 2:** Types of included studies

**Qualitative studies**	36
Qualitative research (case studies)	10
Grey literature (case studies)	9
Multimethod qualitative research	5
Qualitative research (interviews)	5
Grey literature (interviews)	2
Grey literature (multimethod qualitative)	2
Qualitative research (ethnography)	1
Qualitative research (multimethod)	1
Grey literature (qualitative)	1
**Mixed methods**	**15**
Mixed methods research	8
Grey literature (mixed methods)	7
**Quantitative studies**	**8**
Survey analysis	5
Interrupted time series analysis	1
Longitudinal study	1
Controlled before and after study	1
**Literature reviews**	**5**
Systematic review	2
Literature review	2
Grey literature (literature review)	1
**Other studies**	**28**
Descriptive piece	5
Multimethod (literature review and qualitative data)	4
Evaluation	4
Doctoral thesis	3
Grey literature (think tank piece)	2
Feasibility study (development of a tool to help commissioners)	2
Grey literature (opinion piece)	2
Framework development	1
Descriptive news article	1
Briefing paper	1
Grey literature (policy paper)	1
Grey literature (strategy piece)	1
Opinion piece	1
**Total**	**92**

The 30 CMOs developed (representing the final programme theory) are linked back to the initial draft programme theory in Additional file 5 [4, 6, 17, 28, 51–53, 55, 56, 58, 60, 63–101] alongside supporting quotes and, where applicable, substantive theories.

### Category: Steps of the commissioning cycle

Commissioners’ usage of data was sometimes related to specific commissioning activities or steps of the commissioning cycle. Data were used both as a source of evidence to inform commissioning decisions, as well as a tool to stimulate improvements. When deciding commissioning priorities, commissioners considered data to be a credible source of evidence for prioritisation, potentially because they perceived them as providing the most ‘objective’ guidance (CMO 1). Four CMOs related to the ‘monitoring and evaluation’ step of the commissioning cycle, where data were used as a tool by commissioners with the aim of improving clinician and service provider performance. Commissioners shared data (e.g. on referral rates and prescribing costs) with clinicians showing how their performance compared relative to their peers based on beliefs that peer pressure and using other clinicians as a reference point (and the resulting competition) could stimulate improvement. They also believed that clinicians were more receptive to feedback from their peers than commissioners (CMO 2). In contexts where commissioners felt clinicians or service providers did not know their performance was below average and they did not want to be seen as ‘managing performance,’ commissioners shared data with them due to a perception that this could empower clinicians and service providers to come up with their own solutions (CMO 3). If data indicated potential for improvement but commissioners suspected that clinicians required help to achieve these improvements and, as in CMO 3, did not wish to be seen as ‘managing performance’ or judging, commissioners sometimes offered support to outliers and underperformers because they wanted to maintain good relationships by being perceived as supportive (CMO 4). In instances where commissioners shared data with clinicians, linking this data to tailored suggestions for action increased clinician engagement and helped them understand how to make improvements (CMO 5).

### Category: Characteristics of data

The actual and perceived characteristics of data influenced how commissioners used them. ‘Actual’ characteristics relate to objective attributes of data such as being data about health inequalities or being part of a combined dataset. Perceived characteristics of data relate to more subjective factors including beliefs about data, such as commissioners believing data did not reflect local circumstances.

### Contexts promoting the usage of data

The types of data themselves served as a facilitator to commissioners using them (CMOs 8–9, 14, 17, 18–19). For example, commissioners were inclined to use data on health inequalities, which they perceived as useful to achieving an important moral and policy objective (CMO 8). Commissioners were more inclined to use data that were ‘real time’ or recent, since they found them useful for providing immediate support (e.g. to outliers) and enabling speedy decisions, and because they had trust that the data reflect the current situation (CMO 19). Commissioners also wanted access to data showing trends and developments over time, which they could use to monitor and because they perceived this to reduce the risk of drawing false conclusions (CMO 14). Combined datasets, i.e. datasets combining data from different sources such as secondary and primary care enabled commissioners to gain a fuller understanding of the ‘patient journey’ and how and when patients used different care services (CMO 9). These combined datasets facilitated comparisons by providing practices with data to compare themselves with, enabling cross-comparisons with Clinical Commissioning Groups (CCGs) across a regional area, and allowing for comparisons with practices that had a similar profile. These comparisons were facilitated because commissioners could identify multiple datapoints related to a practice or a CCG (e.g. deprivation levels, population factors) thereby allowing them to choose a suitably similar comparator. Commissioners also valued data linked to cost implications, especially the estimated short-term costs of potential interventions, since they were often allocated annual budgets (CMO 17). When commissioners could segment or ‘drill down’ in data, they could create more targeted and tailored commissioning decisions, because they could segment and disaggregate data in multiple ways including by population group, health conditions, and data on service use by ethnic group (CMO 18).

There were also several contexts relating to perceived or more subjective characteristics of data that facilitated their usage (CMOs 6, 7, 13, 20). Where commissioners wished to better understand data, they sometimes supplemented it with qualitative information to increase data validity and gain a fuller and more meaningful understanding (CMO 6). Commissioners sometimes preferred using local data or thought that national or ‘universal’ data (e.g. from trials or research papers) required ‘contextualisation’ (i.e. the process of applying local knowledge to data) or ideally supplementation with local data (CMO 7). Presenting key pieces of data in a succinct, easily digestible manner (e.g. as summaries on a single A4) increased commissioners’ engagement with data (CMO 13). Commissioners were also able to use ‘imperfect’ data such as incomplete data, provided they understood their limitations and this was the only type of data they had access to, since they believed this was better than using no data at all (CMO 20).

### Contexts inhibiting the usage of data

Several characteristics of data inhibited their usage (CMO 10–12, 15–16). If commissioners felt that factors outside of clinicians’ or service providers’ control were impacting benchmarking/variation data, and data did not allow for a ‘like for like’ comparison, they became less inclined to use data because they felt the data were not valid (CMO 10). Similarly, commissioners doubted the credibility of data not presented in an interoperable way, i.e. with consistent definitions, and had difficulty drawing conclusions from them (CMO 11). Feelings of mistrust and a subsequent inclination to use data were also triggered in contexts where (clinical) commissioners perceived data to be in tension with their own knowledge or information from clinicians (CMO 12) or where commissioners suspected that commissioning data were inaccurate or contradictory (CMO 15). If commissioners had access to more data than they could manage, they experienced ‘data overload,’ leaving them frustrated, unable to access (certain) data quickly, and unsure about what to prioritise (CMO 16).

### Category: Commissioners’ capabilities, roles, working environment, and intentions

Commissioners’ capabilities, including their skillsets, capacity for analysis, and understanding of data, as well as their working environment, influenced how they used data.

### Contexts promoting the usage of data

Two CMOs (CMO 22, 24) related to potential interventions that could promote the usage of data: commissioners’ ability to choose data and metrics (e.g. those used to track the progress or uptake of a programme) increased their engagement with data because they were able to choose those they believed were meaningful and valid (CMO 22). Having a ‘data champion’ within the commissioning team to support and promote the usage of data could increase engagement with data and persuade people to use them (CMO 24). In addition, commissioners used data (sometimes selectively) as a source of evidence to persuade others and justify proposals due to a perception that they were an ‘objective’ source of evidence that could increase the legitimacy of proposals (CMO 26). Data were sometimes chosen selectively (rather than systematically) to support what commissioners already wanted or had decided to do prior to looking at evidence (CMO 26).

### Contexts inhibiting the usage of data

If commissioners were subjected to financial pressures, they sometimes chose to make decisions based on little or no evidence (including data) because they felt obliged to prioritise financial issues (CMO 21). Where commissioners lacked the skills to analyse and interpret data, they could not understand it and draw insights from it due to a knowledge gap (CMO 25). Similarly, commissioners were sometimes unable to operationalise data because they had difficulty understanding the drivers of data trends,e.g. the drivers of costs or the reasons behind variation in prescribing rates (CMO 26).

### Category: Interpersonal relationships with and perceptions of external providers

External providers are organisations who provide commissioners with data and/or data analysis including NHS organisations such as commissioning support units (CSUs), academic partners, and private firms such as consultancies and analytics providers. The relationships commissioners had with these external providers influenced how data were used.

### Contexts promoting the usage of data

If commissioners perceived external support as able to provide new or different skills, especially analytical skills, thereby producing new insights, commissioners were more inclined to use the data and outputs produced (CMO 27). The relationships between commissioners and external providers were also key to facilitating data usage. Where commissioners and external providers worked on data production and analysis collaboratively in a way that aligned with the principles of coproduction (e.g. commissioners and external providers having an active and equal relationship, active involvement of commissioners in service design, dialogue between commissioners and external providers), commissioners appeared more inclined to use the outputs (CMO 28). Commissioners who developed relationships with external providers of data (analysis) they perceived to be satisfactory (as evidenced by feelings of trust, closeness, cohesion, etc.) were more likely to use data provided by the external providers (CMO 29).

### Contexts inhibiting the usage of data

If there is a real or perceived divergence of interest or information asymmetry between commissioners and the external providers of data (analysis), commissioners may feel mistrustful (CMO 30). This was informed by the theory of the principal-agent problem, which involves two parties exchanging resources: the principal disposes of resources to an agent, who accepts the resources and is willing to further the interests of the principal (e.g. the principal may give the agent money in exchange for the agent’s skills, or in this case commissioners may hire external consultants) [102]. There is also risk of a potential divergence of interest between the principal and the agent, meaning the principal cannot ensure that the agent will act in their interest [103].

## Discussion

By drawing on 92 studies, we completed the first realist review to focus specifically on the usage of data in primary care commissioning. The resulting 30 CMOs and programme theory offer a novel perspective on the contexts that can facilitate and hinder the usage of data: although commissioners are often eager and willing to use data to inform commissioning decisions, they face a range of challenges that can impede their use, and addressing these will require changes to be made to the data themselves, as well as the manner in which data are presented and shared with commissioners. These CMOs are interrelated, and to increase the usage of data in commissioning it will not be sufficient to address individual CMOs in isolation.

### Comparison to existing literature

Realist research has investigated how policy-makers use evidence and research in countries such as Australia, France, and Canada [49, 104, 105]. Many of the mechanisms identified that promoted and inhibited the usage of evidence (or data) in these studies were similar to those identified in our review, with the actual, ‘objective’ characteristics of evidence being only one factor impacting its usage, in addition to individual, environmental, and organisational factors. A novel contribution of this study is the complexity of outcomes, focussing not only on binary outcomes relating to whether data were used or not but also on more nuanced ones such as using data in conjunction with other forms of evidence or employing strategies to use ‘imperfect’ data.

Non-realist studies of evidence-based decision making in policy have correspondingly found that the usage of evidence in decision-making is a complex, context dependent process and that evidence is often underutilised. The findings of this study have confirmed many of the findings of non-realist research on evidence use in policy-making, e.g. that a gulf or disconnect between decision-makers and researchers (or evidence providers) can prevent evidence from being used [106], or that a lack of time and resources inhibits evidence use [107]. Non-realist research on evidence-use in policy-making has concluded that there is a need for context specific research about the best approaches for incorporating research evidence into decision making [106] and that further research is needed on how and why different types of evidence are used in decision-making [107]. This study has built on this by investigating in more detail the contexts impacting the usage of data, as well as providing insights on specific types of data such as variation data or combined datasets.

The findings of this study are concordant with findings that interventions to increase commissioners’ usage of evidence by providing them with more evidence or embedding researchers in commissioning organisations have not always been successful, confirming that increased access to evidence and its producers is not necessarily sufficient to increase its uptake and that more complex contextual factors are at play. A study evaluating whether access to a demand-led evidence briefing service improved the use of research evidence by commissioners found that this did not improve the uptake and use of research evidence compared to less targeted and intensive alternatives [14]. An evaluation of a ‘researcher in residence’ model in three organisations, including a CCG, concluded that it had potential to produce knowledge that could be used in practice, but challenges remained, including how best to embed researchers in their host environment and the development of relationships with commissioners [108].

### Strengths and limitations

The strengths of this study include its novel contribution to the literature, since it is, to the best of our knowledge, the first study to synthesise secondary literature on the usage of data as a form of evidence in primary care commissioning decisions. The application of a realist lens to this topic has provided an elucidation of contexts and their inherent interrelatedness that promote and inhibit the usage of data in primary care commissioning, issues that had received limited attention in the existing literature. This study has also provided insights on the contexts affecting the usage of specific types of data such as variation data or combined datasets. Understanding the latter is particularly pertinent in light of NHS policy to support more integrated commissioning in ICBs and ICSs, since these will have to commission ‘joined up’ health and care services across, e.g. secondary and primary care. A large amount of diverse secondary literature (including grey literature) was synthesised, thereby potentially increasing the validity of the findings.

A limitation of this study is that synthesised studies were largely atheoretical, and there is not one specific NHS-articulated programme theory underpinning the usage of data in primary care commissioning. It was therefore challenging to create an initial programme theory and it was not possible to do so in a realist format, which could have facilitated and accelerated CMO development. In addition, there are several limitations inherent to any realist review, including that more informal or ‘off the record’ information on contextual factors such as interpersonal relationships or power struggles may not be documented in studies [109]. A realist review is not reproducible in the same sense as a Cochrane review, but quality assurance can be provided by researchers being explicit about review methods [109]. While we have attempted to make our methods and reasoning transparent, the CMOs developed may have been different had this study been conducted by a commissioner or another policy-maker.

### Implications for practice and policy

Based on the CMOs developed, we have developed several policy recommendations that could potentially facilitate and increase commissioners’ usage of data. As previously mentioned, our CMOs are interrelated, and it is unlikely that implementing a single recommendation or addressing a single CMO can effect change. In addition, each commissioning organisation likely has a different baseline in terms of how (often) data are used, meaning not every recommendation is applicable to every organisation. Therefore, the following recommendations are to be understood as something we recommend commissioners consider, as addressing these will likely help increase the chances that data will be used in commissioning, but the applicability of recommendations will vary:

- Increase collaboration with the external providers of data: when commissioners can develop relationships and collaborate with the external providers of data, they can better communicate their needs and co-produce relevant evidence. This can also facilitate feelings of trust and make commissioners less wary of external providers’ motivations, thereby making them more likely to use the data.
- Implement a ‘data champion’ in each commissioning team: commissioners are receptive to messaging and leadership from their peers around data. Having a data champion in their commissioning team who they view as an equal can increase engagement with data.
- Give commissioners access to up-to-date, locally relevant, and manipulatable datasets: commissioners want access to data they can perform their own analyses on, rather than static data, as this can make the data more useful and meaningful to them.
- Improve the availability of meaningful integrated data: with the advent of ICSs, integrated, combined datasets (including combining data across different types of care, e.g. primary and secondary care as well as data on social determinants of health e.g. inequality data) are more important than ever. Being able to see the full ‘patient journey’ and considering how non-medical factors impact health outcomes can enable customised commissioning.
- Define skills and competencies for commissioners and provide training: commissioners come from a wide range of academic backgrounds and prior work experiences. Some commissioners lack the capability to perform or interpret data analysis. Defining the skills that are expected of commissioners in terms of data analysis and providing training could increase engagement with data.

### Implications for research

In mid-2022, CCGs were dissolved and replaced by ICSs, with ICBs of each ICS taking on a range of commissioning responsibilities, including primary care services [9]. Several recent NHS policy documents have outlined the importance of ICSs using data to deliver population health improvements, address health inequalities, and develop ICS-wide fully linked datasets from data that are still mostly held separately by individual services and their commissioners [110]. Future research could test the applicability of the CMOs developed in this study to ICSs, in particular CMO 9 (combined datasets) and CMO 12 (interoperability), and refine and develop new CMOs as required.

Given the government’s ‘National Data Strategy’ and its commitment to using ‘better data’ for ‘better decision making’ to deliver more tailored and efficient policies and make savings [111], future research could assess, compare, and contrast how similar areas of policy-making (such as social care, education, or the justice system) use data to inform the planning and commissioning of services. By identifying common barriers and facilitators to using data in policy-making as well as methods to overcoming the concomitant challenges, policy-makers could learn from each other and improve their own practice of evidence use.

## Conclusions

Considering the NHS’s increased ambition, commitment to, and investment in using data to inform commissioning, combined with the financial pressures stemming from an ageing population facing increasingly complex health challenges, the need to understand how data can be used effectively to inform commissioning is greater than ever. One encouraging finding from this research is that commissioners do value data as a source of evidence to inform their decision-making, perceiving data to be ‘rational,’ persuasive, and useful if presented and used correctly. Although commissioners are often eager and willing to use data to inform commissioning decisions, they face a range of challenges that can impede their use, and addressing these where they occur will require changes to be made to the data themselves, as well as the way data are presented and shared with commissioners. In addition, increasing commissioners’ trust in the quality of data and strengthening their relationships with and trust in those who provide them with data could facilitate data usage. There is evidence of a disconnect between how NHS policy-makers and providers of data believe the data are used in commissioning and how they are actually used, and greater collaboration and exchange between them and commissioners could facilitate better usage.

## Supplementary Information

**Additional file 1.** Detailed overview of 5-step review process.

**Additional file 2.** Emerging CMO configurations.

**Additional file 3.** Literature research.

**Additional file 4.** Table of included studies.

**Additional file 5.** Final programme theory.

## Data Availability

Datasets used in the review are available from the corresponding author upon reasonable request.

## Abbreviations

CCGs: Clinical Commissioning Groups
CSUs: Commissioning support units
CMO: Context-mechanism-outcome
ICBs: Integrated care boards
ICSs: Integrated care systems
NHS: National Health Service
RAMESES: Realist And MEta-narrative Evidence Syntheses: Evolving Standards
