# Supplementary material for: The usage of data in NHS primary care commissioning: a realist review
Source: BMC Med. 2023 Jul 3;21:236. doi: 10.1186/s12916-023-02949-w (PMC10318817; doi:10.1186/s12916-023-02949-w)
Supplement: Supplementary file 1 — Additional file 1. Detailed overview of 5-step review process. [file 12916_2023_2949_MOESM1_ESM.docx]

### Step 1: Locate existing theories

We developed initial programme theory through an exploratory literature search (additional file 3), ultimately identifying 15 studies, and informal discussions. Three informal discussions with a former and a current NHS commissioner as well as with someone who worked in public health in the NHS were completed, exploring how they used evidence to inform decision-making and the contextual factors surrounding this. We were not able to find a ‘theorised’ version of the NHS commissioning cycle to help build programme theory: a policy document containing an overview of the types of data that could be used to inform commissioning as well as the NHS’s own official ‘commissioning cycle’ outlining nine steps related to commissioning were also identified, but these did not provide guidance on the barriers or facilitators to using data or the contexts related to their usage. Initial programme theory was built by identifying barriers and facilitators to using data to inform primary care commissioning contained in the 15 studies and informal conversations. These are summarised by theme and presented as potential (but incomplete and emerging) CMO configurations in additional file 2.

### Step 2: Search for evidence

A broad and interdisciplinary formal literature search, designed with the help of an information specialist (NR), was completed to capture a wide range of studies (additional file 3). Several versions of more and less specific stings were trialled and the results compared. Ultimately, we developed a search string containing four topic areas (data, commissioning, NHS, and primary care). We searched the following seven databases in March 2019: CINAHL, Embase, Health Management Information Consortium, NIHR-HTA Database, ProQuest Dissertations & Theses Global‎, Scopus, and Web of Science. We completed grey literature searches via the NHS Digital website, Open Grey, Trip database, the King’s Fund website, and NICE evidence search.

An additional search in October 2019 focussed on modifying the search string to incorporate the phrase ‘evidence based commissioning.’ During the initial database search, several useful and theory-rich studies containing this phrase in the abstract were identified. This search only returned a small number of studies, and the studies meeting the inclusion criteria had already been identified in the main database search in March 2019. In March 2022, an updated database search to identify studies that had been published between March 2019 and 2022 was completed. In addition, some studies were identified via reference linking.

### Step 3: Select studies

The selection of studies focussed on the ability of articles to contribute to and refine the programme theory. We imported the studies identified in step 2 into Rayyan, a systematic review software. We screened titles and abstracts and selected them for full-text review if they met the inclusion criteria outline in table 1.

Table 1 Inclusion and exclusion criteria

| **Inclusion criteria** | **Exclusion criteria** |
| --- | --- |
| The article is written in English. | The article is written in a language other than English. |
| The article describes how data have been used, either alone or in conjunction with other forms of evidence, to make, inform, or influence decisions about commissioning primary care services within the NHS in England. | The article does not describe how data have been used to make, inform, or influence decisions about commissioning primary care services within the NHS in England. |
| The article describes how data were actually used to make, inform, or influence decisions about commissioning primary care services within the NHS in England. | The article describes how data could or should be used to make, inform, or influence decisions about commissioning primary care services within the NHS in England. |

Many of the studies were about commissioning as a whole in the NHS rather than the commissioning of a specific type of service e.g. secondary or primary care commissioning. Therefore, in addition to studies focusing solely on the commissioning of primary care services, studies about commissioning pathways or services that were in part located in primary care were included. In addition, some studies focussed on commissioning at an organisational level, e.g. on a specific CCG or Primary Care Trust (PCT). Provided the organisation was one that commissioned, inter alia, primary care, the study was included. No date restriction was applied to the database searches, however since the provider-purchaser split in the NHS occurred in 1991, there were no articles predating this.

During the title and abstract screening stage, a random 10% of the studies were given to a second reviewer (MA) to ensure the inclusion criteria were being applied consistently. MA and AJ (the lead author) discussed any disagreements and ambiguities and refined the inclusion criteria as well as our approach to screening as a result to achieve more consistency. Following the title and abstract screening, full text screening commenced. We used three criteria to determine if an article should be included: relevance, rigour, and trustworthiness. According to Pawson, data (or studies) are relevant if they contribute to programme theory, i.e. if they help corroborate, refute or refine its different aspects. Assessment of rigour is conducted by analysing whether the methods used to generate data are credible and trustworthy. Realist researchers also use the criterion of trustworthiness to assess data quality by applying the following considerations: (i) check if the articles outline the methods for data collection, and are thereby unlikely to be fabricated, (ii) treat articles with scepticism if the methods for obtaining data are not stated, (iii) attempt to find more than one source of data to support each aspect of programme theory.

No restriction to the type of study was applied: when conducting a realist synthesis, researchers should consider a range of empirical evidence and assess its value in terms of contribution to programme theory development rather than following a ‘methodological hierarchy of evidence’. Once more, a random 10% of the articles were given to the same second reviewer for full-text screening as per the inclusion criteria to ensure consistency. We discussed inconsistencies and disagreements between our screening decisions and resolved these through discussion and refinement of the screening criteria.

### Step 4: Extract and organise data

Following full-text screening, we imported the included studies into NVivo 12 (a qualitative data management software package) for coding. The key characteristics of the 92 included studies are shown in additional file 4.

### Step 5: Synthesise the evidence according to a realist logic of analysis

During the first read of the included studies, we coded all relevant concepts and ideas (without necessarily following a realist logic of analysis), as well as any substantive theories mentioned. Some of these codes were initially very granular and would later be grouped into bigger categories, bringing in additional substantive theory or concepts from the broader academic literature where possible.

After reading all of the articles once, we re-read studies that were particularly relevant to the programme theory, i.e. those providing the richest insights, first. Several forms of reasoning were used to identify contexts, mechanisms, and outcomes, namely induction, deduction, retroduction, and abduction, defined as follows:

| **Induction:** the process of generating theory from evidence or deriving conclusions from observations  **Deduction:** the testing of theory against evidence or testing theory to see whether associations match expectations  **Retroduction:** is a form of inference that aims to identify the hidden causal forces that lie behind patterns or regularities to discover what produces them. It requires the usage of inductive and deductive logic, as well as insights or hunches, and is based on a belief that understanding causation can not only be based on only observable evidence.  **Abduction:** refers to ‘inference to the best explanation’ and involves a process of examining evidence and developing hunches or ideas about the causal factors linked to that evidence: it is the inventive thinking required to imagine the existence of the mechanisms tested via retroduction. The process of abduction allows empirical data and ideas to be re-described using theoretical concepts or frameworks, and is often applied to incomplete data and relies on theories as mediators – for example, if a researcher observes outcome X, and can reason that Y is a prerequisite for X, there is reason to expect Y to be true, i.e. inference to the most plausible explanation. |
| --- |

Once draft CMOs had been developed, we used several forms of reasoning as suggested by Pawson, namely juxtaposition, reconciliation, consolidation, and situating, to further refine and develop the CMOs:

| **Juxtaposition:** used to place two or more things or evidence fragments together and suggest a link between them – for example, one study can provide data to make sense of an outcome noted in another study.  **Reconciliation:** identifying differences which explain apparently contradictory findings, or discovering contextual differences that show how opposing outcomes were reached  **Consolidation:** building ‘multi-faceted explanations of success.’ For example, if there are two successful outcomes that are the result of different mechanisms and contexts, consolidation allows the researcher to consolidate the results into a multi-faceted explanation of success (usually linked to a larger and more complex CMO).  **Situating:** the more granular situation of rival theories (‘this mechanism in context A, that one in context B’). |
| --- |

We began by coding outcomes (proximal or final): once an outcome relevant to the programme theory had been identified in a paper, this was linked to contexts where possible and subsequently to mechanisms. In line with other realist research on evidence use, we considered not only the actual usage (or not) of data, but also the intention to (not) use data as an outcome when constructing CMOs. Such proximal outcomes include factors such as perceptions about the value of research, knowledge, and skills that make the final outcome, i.e. the actual (non-) usage of evidence, more or less likely. Sometimes it was possible to identify a full CMO within one study, but more often we had to link various contexts, mechanisms, and outcomes across several different studies.
