# Supplementary material for: The usage of data in NHS primary care commissioning: a realist review
Source: BMC Med. 2023 Jul 3;21:236. doi: 10.1186/s12916-023-02949-w (PMC10318817; doi:10.1186/s12916-023-02949-w)
Supplement: Supplementary file 2 — Additional file 2. Emerging CMO configurations. [file 12916_2023_2949_MOESM2_ESM.docx]

| **Theme** | **Summary of key points in literature/information from informal discussions** | **Potential incomplete CMO configurations** |
| --- | --- | --- |
| **Financial considerations** | - Financial constraints can temper the direct influence of research evidence on decision making - Not being able to see the financial implications of data is a barrier to using data - If commissioners must deliver a balanced financial plan sometimes commissioning decisions based on little or no evidence are funded - A core role of a commissioner is to ensure you don’t overspend, constant pressure to make financial savings (informal discussions) | - If commissioners feel pressure to make financial savings, they may make commissioning decisions not necessarily based on evidence to achieve this - If commissioners cannot see the financial implications linked to data, they may be less included to use them |
| **Political model of data usage** | - This is a political model of data usage: research is ammunition for the side that finds its conclusions supportive, and is used to ‘neutralise opponents’ - Sometimes purposely sought information since they were told to take a particular course of action, e.g. national directives - ‘Cherry picking’ of data does happen – sometimes the idea comes first, and then data is sought to substantiate this (informal discussions) | - Commissioners may choose data selectively to support what they want to do |
| **Collaborations with external providers of data (analysis)** | - A primary care commissioner critiqued having been ‘given’ rather than ‘chosen’ a software tool to help identify individuals at higher risk of using healthcare resources e.g. hospital beds by its developers, and that they had difficulty interpreting and contextualising its outputs - Collaborations with external providers can work well or not depending on several factors including the relationships between commissioners and external providers and trust - Commissioners are not always happy with the data their receive from external providers, and sometimes feel they don’t receive the information they need | - The relationship and communication between commissioners and external providers of data (analysis) is decisive in determining whether the data will be used |
| **Knowledge of clinical vs. non-clinical commissioners** | - GPs may have different knowledge of data and information and patients than other commissioners and may challenge data based on their clinical knowledge | - If GP commissioners believe there is a contradiction between data and their experience, they may challenge them and be less inclined to use them |
| **Triangulation and combining of data sources** | - Triangulation of different benchmarking data was found to be helpful in identifying areas for action - Commissioners want to have access to combined datasets, especially at the patient level | - Combining and triangulating data sources may facilitate the usage of data |
| **Presentation of data, data overload** | - Commissioners have extensive access to evidence including national and regional directives, meeting papers, business cases, reports, patient satisfaction surveys, guidelines, pathways, and performance, activity, financial and referral data. To capture commissioners’ attention, these documents often had a summary of no more than one side of A4 with clearly bulleted action points | - The format in which data are presented may facilitate or hinder their usage |
| **Local vs. national data** | - Local data often trumped national or research-based information and local evaluations were seen as helpful in directly answering commissioners’ questions - Commissioners consider local data to be an important source of evidence, sometimes this was because a local population has distinctive features | - Local data may trump national data in certain contexts |
| **Data vs. information** | - Commissioners sometimes have challenges operationalising data | - If commissioners cannot operationalise data, they cannot use them |
| **Variation data** | - Can be used in myriad ways to drive improvement, including by stimulating competition and peer-pressure, but may also lead commissioners to offer support to practices and/clinicians | - Commissioners may use variation data in different ways depending on context |
| **Data quality** | - Some commissioners felt the data they had access to was of poor quality or untimely and that access to real-time and more timely data would be beneficial | - Poor quality and untimely data are unlikely to be used by commissioners |
