# Supplementary material for: The usage of data in NHS primary care commissioning: a realist review
Source: BMC Med. 2023 Jul 3;21:236. doi: 10.1186/s12916-023-02949-w (PMC10318817; doi:10.1186/s12916-023-02949-w)
Supplement: Supplementary file 3 — Additional file 3. Literature research. [file 12916_2023_2949_MOESM3_ESM.docx]

**Exploratory literature search (review step 1):**

The exploratory literature search consisted of a keyword search (‘Commissioning’ AND ‘Data’ AND ‘NHS’ AND ‘primary care’) carried out in February 2020 in the MEDLINE/PubMed databases as well as Google Scholar, in addition to a Google search to locate grey literature.

**Initial database search – March 2019**

| Grey literature searches – all searched in the spring of 2019 | | | |
| --- | --- | --- | --- |
| Website | **Source name** | **Hits** | **Search strategy** |
| <https://digital.nhs.uk/> | NHS Digital | 200 | Go to the website for NHS Digital (https://digital.nhs.uk/) and enter the following search string in the box on the main page:  commissioning AND (data OR surveillance OR insights OR intelligence) AND ("primary care" OR "primary health" OR "general practice")   When it displays the search results, there is a box on the far left that says 'document type' - select "publications" here |
| <http://www.opengrey.eu/> | Open Grey | 33 | Go to http://www.opengrey.eu/ and enter the following search string:  (NHS OR "national health service") AND (data OR surveillance OR insights OR intelligence) AND ("primary care" OR "primary health" OR "general practice") |
| <https://www.tripdatabase.com/> | TRIP Database | 324 | Go to the TRIP database (https://www.tripdatabase.com/) enter the following under search: commissioning AND primary care AND data AND NHS  On the right hand side of the page, you will be able to select certain document types. Please select the following: Evidence-based synopses (284 documents), primary research (40 documents) (not key primary research, this is a separate category) |
| <https://www.kingsfund.org.uk/> | The King's Fund | 71 | Go to Google.co.uk, and type in the following search string:  .pdf site:https://kingsfund.org.uk commissioning AND (data OR surveillance OR insights OR intelligence) AND ("primary care" OR "primary health" OR "general practice")  This will search for PDFs (i.e. reports only), using Google to search the King's Fund site |
| <https://www.evidence.nhs.uk/> | NICE Evidence search | 387 | Search string: (NHS OR "national health service") AND (data OR insights OR intelligence) AND ("primary care" OR "primary health" OR "general practice") AND (commissioning). Filter: focusing on Area of interest - Commissioning and Management. It is not possible to export these files into Rayyan/Ref Works including the summary description, so I had to review them manually on the website. This hits may contain duplicates and included studies for full-text screening will be included under studies identified through hand-searching on the PRISMA diagram. |

| Database searches | | | | | | |
| --- | --- | --- | --- | --- | --- | --- |
| Database name | **Hits** | **Fields searched** | **Date searched** | **Search syntax** | **Notes** | |
| CINAHL (Cumulative Index to Nursing and Allied Health Literature) | 295 | Abstract | 30/03/2019 | S1: AB (Data OR Insights OR Statistics OR Business Analy* OR business intelligence) Search modes - Boolean/Phrase  S2: AB (NHS OR National Health Service OR Public Health England OR Department of Health OR English healthcare OR English Health Care) Search modes - Boolean/Phrase  S3: AB(competitive tendering OR fundholding OR fund-holding OR strategic contracting OR strategic buying OR procurement OR purchas* OR contracting out OR gpfh OR Primary Care Groups OR PCG* OR Primary Care Trust* OR PCT* OR Clinical Commissioning Group* OR CCG* OR commissioning OR commissioner* OR Assessing needs OR reviewing service provisions OR deciding priorities OR designing services OR "shaping structure of supply" OR "planning capacity and managing demand" OR supporting patient choice OR managing performance OR "seeking public and patient views" OR Needs assessment OR planning OR Market management OR Market development OR decommissioning OR disinvestment OR performance management OR procuring services OR "monitoring and evaluation") Search modes - Boolean/Phrase  S4: AB(Primary Care OR Primary healthcare OR Primary Health Care OR Primary Health-Care OR Primary Health OR phc OR general practi*)  Final search: (S1 AND S2 AND S3 AND S4) |  | |
| Embase | 1,710 | Keyword (mp, mp,in. for NHS terms) | 30/03/2019 | (Primary Care or Primary healthcare or Primary Health Care or Primary Health-Care or Primary Health or phc or general practi*).mp. [mp=title, abstract, heading word, drug trade name, original title, device manufacturer, drug manufacturer, device trade name, keyword, floating subheading word, candidate term word] AND (Data or Insights or Statistics or Business Analy* or business intelligence).mp. [mp=title, abstract, heading word, drug trade name, original title, device manufacturer, drug manufacturer, device trade name, keyword, floating subheading word, candidate term word] AND (NHS or National Health Service or Public Health England or Department of Health or English healthcare or English Health Care).mp,in. AND (competitive tendering or fundholding or fund-holding or strategic contracting or strategic buying or procurement or purchas* or contracting out or gpfh or Primary Care Groups or PCG* or Primary Care Trust* or PCT* or Clinical Commissioning Group* or CCG* or commissioning or commissioner* or Assessing needs or reviewing service provisions or deciding priorities or designing services or "shaping structure of supply" or "planning capacity and managing demand" or supporting patient choice or managing performance or "seeking public and patient views" or Needs assessment or planning or Market management or Market development or decommissioning or disinvestment or performance management or procuring services or "monitoring and evaluation").mp. [mp=title, abstract, heading word, drug trade name, original title, device manufacturer, drug manufacturer, device trade name, keyword, floating subheading word, candidate term word] |  |  |
| Health Management Information Consortium | 678 |  | 30/03/2019 | (Primary Care or Primary healthcare or Primary Health Care or Primary Health-Care or Primary Health or phc or general practi*).mp. AND (Data or Insights or Statistics or Business Analy* or business intelligence).mp. AND (NHS or National Health Service or Public Health England or Department of Health or English healthcare or English Health Care).mp. AND (competitive tendering or fundholding or fund-holding or strategic contracting or strategic buying or procurement or purchas* or contracting out or gpfh or Primary Care Groups or PCG* or Primary Care Trust* or PCT* or Clinical Commissioning Group* or CCG* or commissioning or commissioner* or Assessing needs or reviewing service provisions or deciding priorities or designing services or "shaping structure of supply" or "planning capacity and managing demand" or supporting patient choice or managing performance or "seeking public and patient views" or Needs assessment or planning or Market management or Market development or decommissioning or disinvestment or performance management or procuring services or "monitoring and evaluation").mp. | Search run by Nia Roberts on 28/03/2019 (students do not have access). Coverage of the database = HMIC Health Management Information Consortium 1979-January 2019 |  |
| NIHR-HTA Database | 25 | Any field, HTA | 30/03/2019 | (data OR insights OR statistics) AND (nhs OR "national health service") AND ("primary care" OR "primary health" OR "primary healthcare" OR phc OR "general practi*") AND (commission* OR ccg OR need* OR demand* OR planning OR capaciy OR performance OR market OR decommission* OR disinvest* OR procur* OR monitor*) |  |  |
| ProQuest Dissertations & Theses Global‎ | 339 | Abstract | 27/03/2019 | S1 AND S2 AND S3 AND S4  S1  ab("Data" OR "Insights" OR "Statistics" OR "Business Analy*" OR "business intelligence")   S2  ab(NHS OR "National Health Service" OR "Public Health England" OR "Department of Health" OR "English healthcare" OR "English Health Care")  S3  ab("competitive tendering" OR fundholding OR fund-holding OR "strategic contracting" OR "strategic buying" OR procurement OR purchas* OR "contracting out" OR gpfh OR "Primary Care Groups" OR "PCG*" OR "Primary Care Trust*" OR "PCT*" OR "Clinical Commissioning Group*" OR "CCG*" OR "commissioning" OR commissioner* OR “Assessing needs" OR "reviewing service provisions" OR "deciding priorities" OR "designing services" OR "shaping structure of supply" OR "planning capacity and managing demand" OR "supporting patient choice" OR "managing performance" OR "seeking public and patient views" OR "Needs assessment" OR "planning" OR "Market management" OR "Market development" OR decommissioning OR disinvestment OR "performance management" OR "procuring services" OR "monitoring and evaluation" )  S4  ab("Primary Care" OR "Primary healthcare" OR "Primary Health Care" OR "Primary Health-Care" OR "Primary Health" OR phc OR "general practi*") | Limit applied is English only. All databases selected |  |
| Scopus | 980 | Title, abstract, key words | 30/03/2019 | ( TITLE-ABS-KEY ( nhs OR "National Health Service" OR "Public Health England" OR "Department of Health" OR "English healthcare" OR "English Health Care" ) ) AND ( TITLE-ABS-KEY ( "competitive tendering" OR fundholding OR fund-holding OR "strategic contracting" OR "strategic buying" OR procurement OR purchas* OR "contracting out" OR gpfh OR "Primary Care Groups" OR "PCG*" OR "Primary Care Trust*" OR "PCT*" OR "Clinical Commissioning Group*" OR "CCG*" OR "commissioning" OR commissioner* OR "Assessing needs" OR "reviewing service provisions" OR "deciding priorities" OR "designing services" OR "shaping structure of supply" OR "planning capacity and managing demand" OR "supporting patient choice" OR "managing performance" OR "seeking public and patient views" OR "Needs assessment" OR "planning" OR "Market management" OR "Market development" OR decommissioning OR disinvestment OR "performance management" OR "procuring services" OR "monitoring and evaluation" ) ) AND ( TITLE-ABS-KEY ( "Primary Care" OR "Primary healthcare" OR "Primary Health Care" OR "Primary Health-Care" OR "Primary Health" OR phc OR "general practi*" ) ) AND ( TITLE-ABS-KEY ( "Data" OR "Insights" OR "Statistics" OR "Business Analy*" OR "business intelligence" ) ) AND ( LIMIT-TO ( LANGUAGE , "English" ) ) |  |  |
| Web of Science | 352 | TS= Topic |  | (TS=("Data"OR"Insights"OR"Statistics"OR"Business Analy*"OR"business intelligence")) *AND*LANGUAGE: (English) AND (TS=(NHS OR "National Health Service" OR "Public Health England" OR "Department of Health" OR "English healthcare" OR "English Health Care")) AND LANGUAGE: (English) AND (TS=("competitive tendering" OR fundholding OR fund-holding OR "strategic contracting" OR "strategic buying" OR procurement OR purchas* OR "contracting out" OR gpfh OR "Primary Care Groups" OR "PCG*" OR "Primary Care Trust*" OR "PCT*" OR "Clinical Commissioning Group*" OR "CCG*" OR "commissioning" OR commissioner* OR “Assessing needs" OR "reviewing service provisions" OR "deciding priorities" OR "designing services" OR "shaping structure of supply" OR "planning capacity and managing demand" OR "supporting patient choice" OR "managing performance" OR "seeking public and patient views" OR "Needs assessment" OR "planning" OR "Market management" OR "Market development" OR decommissioning OR disinvestment OR "performance management" OR "procuring services" OR "monitoring and evaluation")) AND LANGUAGE: (English) AND (TS=("Primary Care" OR "Primary healthcare" OR "Primary Health Care" OR "Primary Health-Care" OR "Primary Health" OR phc OR "general practi*")) AND LANGUAGE: (English) |  |  |

**Additional search – October 2019**

| Database | Search string | Fields searched | Results | Notes |
| --- | --- | --- | --- | --- |
| CINAHL | "evidence based commissioning" OR "evidence-based commissioning" | Abstract | 4 | Search modes: Boolean/phrase |
| Embase | ("evidence based commissioning" or "evidence-based commissioning").mp. | [mp=title, abstract, heading word, drug trade name, original title, device manufacturer, drug manufacturer, device trade name, keyword, floating subheading word, candidate term word] | 16 |  |
| ProQuest Dissertations & Theses Global | ab("evidence-based commissioning") OR ab("evidence based commissioning") | Abstract | 2 |  |
| Scopus | TITLE-ABS-KEY ( "evidence based commissioning" ) OR TITLE-ABS-KEY ( "evidence-based commissioning" ) | Article title, Abstract, Keywords | 16 |  |
| Web of Science | ("evidence-based commissioning") OR ("evidence based commissioning") | Topic | 8 |  |
| NIHR-HTA Database | ("evidence-based commissioning") OR ("evidence based commissioning") | Any field | 0 |  |

**Updated search – March 2022**

| Name of database | Date searched | Results | Fields searched | Query string |
| --- | --- | --- | --- | --- |
| Scopus | 02/03/2022 | 136 | Title, abstract, key words | ( TITLE-ABS-KEY ( "Data" OR "Insights" OR "Statistics" OR "Business Analy*" OR "business intelligence" ) AND TITLE-ABS-KEY ( nhs OR "National Health Service" OR "Public Health England" OR "Department of Health" OR "English healthcare" OR "English Health Care" ) AND TITLE-ABS-KEY ( "competitive tendering" OR fundholding OR fund-holding OR "strategic contracting" OR "strategic buying" OR procurement OR purchas* OR "contracting out" OR gpfh OR "Primary Care Groups" OR "PCG*" OR "Primary Care Trust*" OR "PCT*" OR "Clinical Commissioning Group*" OR "CCG*" OR "commissioning" OR commissioner* OR "Assessing needs" OR "reviewing service provisions" OR "deciding priorities" OR "designing services" OR "shaping structure of supply" OR "planning capacity and managing demand" OR "supporting patient choice" OR "managing performance" OR "seeking public and patient views" OR "Needs assessment" OR "planning" OR "Market management" OR "Market development" OR decommissioning OR disinvestment OR "performance management" OR "procuring services" OR "monitoring and evaluation" ) AND TITLE-ABS-KEY ( "Primary Care" OR "Primary healthcare" OR "Primary Health Care" OR "Primary Health-Care" OR "Primary Health" OR phc OR "general practi*" ) ) AND ( LIMIT-TO ( PUBYEAR , 2022 ) OR LIMIT-TO ( PUBYEAR , 2021 ) OR LIMIT-TO ( PUBYEAR , 2020 ) OR LIMIT-TO ( PUBYEAR , 2019 ) ) AND ( LIMIT-TO ( LANGUAGE , "English" ) ) |
| Web of Science | 02/03/2022 | 145 |  | (((TS=("Data" OR "Insights" OR "Statistics" OR "Business Analy*" OR "business intelligence")) AND TS=(NHS OR "National Health Service" OR "Public Health England" OR "Department of Health" OR "English healthcare" OR "English Health Care")) AND TS=("competitive tendering" OR fundholding OR fund-holding OR "strategic contracting" OR "strategic buying" OR procurement OR purchas* OR "contracting out" OR gpfh OR "Primary Care Groups" OR "PCG*" OR "Primary Care Trust*" OR "PCT*" OR "Clinical Commissioning Group*" OR "CCG*" OR "commissioning" OR commissioner* OR “Assessing needs" OR "reviewing service provisions" OR "deciding priorities" OR "designing services" OR "shaping structure of supply" OR "planning capacity and managing demand" OR "supporting patient choice" OR "managing performance" OR "seeking public and patient views" OR "Needs assessment" OR "planning" OR "Market management" OR "Market development" OR decommissioning OR disinvestment OR "performance management" OR "procuring services" OR "monitoring and evaluation")) AND TS=("Primary Care" OR "Primary healthcare" OR "Primary Health Care" OR "Primary Health-Care" OR "Primary Health" OR phc OR "general practi*") |
| CINAHL | 15/03/2022 | 89 |  | AB ( Data OR Insights OR Statistics OR Business Analy* OR business intelligence ) AND AB ( NHS OR National Health Service OR Public Health England OR Department of Health OR English healthcare OR English Health Care ) AND AB ( competitive tendering OR fundholding OR fund-holding OR strategic contracting OR strategic buying OR procurement OR purchas* OR contracting out OR gpfh OR Primary Care Groups OR PCG* OR Primary Care Trust* OR PCT* OR Clinical Commissioning Group* OR CCG* OR commissioning OR commissioner* OR Assessing needs OR reviewing service provisions OR deciding priorities OR designing services OR "shaping structure of supply" OR "planning capacity and managing demand" OR supporting patient choice OR managing performance OR "seeking public and patient views" OR Needs assessment OR planning OR Market management OR Market development OR decommissioning OR disinvestment OR performance management OR procuring services OR "monitoring and evaluation" ) AND AB ( Primary Care OR Primary healthcare OR Primary Health Care OR Primary Health-Care OR Primary Health OR phc OR general practi* ) |
| Embase (see below) | 02/03/2022 | 172 |  |  |
| ProQuest Dissertations & Theses Global‎ (see below) | 02/03/2022 | 8 |  |  |

**Embase**

| [# ▲](https://ovidsp.dc1.ovid.com/ovid-b/ovidweb.cgi?&S=KLKPFPECIIACBOOAKPNJMEOIOBPNAA00&Sort+Sets=descending) | Searches | Results |
| --- | --- | --- |
|  |  |  |
| 1 | (Primary Care or Primary healthcare or Primary Health Care or Primary Health-Care or Primary Health or phc or general practi*).mp. [mp=title, abstract, heading word, drug trade name, original title, device manufacturer, drug manufacturer, device trade name, keyword heading word, floating subheading word, candidate term word] | 400216 |
|  |  |  |
| 2 | limit 1 to (english language and yr="2019 - 2022") | 63891 |
|  |  |  |
| 3 | (Data or Insights or Statistics or Business Analy* or business intelligence).mp. [mp=title, abstract, heading word, drug trade name, original title, device manufacturer, drug manufacturer, device trade name, keyword heading word, floating subheading word, candidate term word] | 6690125 |
|  |  |  |
| 4 | limit 3 to (english language and yr="2019 - 2022") | 1419972 |
|  |  |  |
| 5 | (NHS or National Health Service or Public Health England or Department of Health or English healthcare or English Health Care).mp. [mp=title, abstract, heading word, drug trade name, original title, device manufacturer, drug manufacturer, device trade name, keyword heading word, floating subheading word, candidate term word] | 131721 |
|  |  |  |
| 6 | limit 5 to (english language and yr="2019 - 2022") | 19892 |
|  |  |  |
| 7 | (competitive tendering or fundholding or fund-holding or strategic contracting or strategic buying or procurement or purchas* or contracting out or gpfh or Primary Care Groups or PCG* or Primary Care Trust* or PCT* or Clinical Commissioning Group* or CCG* or commissioning or commissioner* or Assessing needs or reviewing service provisions or deciding priorities or designing services or "shaping structure of supply" or "planning capacity and managing demand" or supporting patient choice or managing performance or "seeking public and patient views" or Needs assessment or planning or Market management or Market development or decommissioning or disinvestment or performance management or procuring services or "monitoring and evaluation").mp. [mp=title, abstract, heading word, drug trade name, original title, device manufacturer, drug manufacturer, device trade name, keyword heading word, floating subheading word, candidate term word] | 724492 |
|  |  |  |
| 8 | limit 7 to (english language and yr="2019 - 2022") | 124383 |
|  |  |  |
| 9 | 2 and 4 and 6 and 8 | 172 |

**ProQuest**

| **S12** | [ab("Data" OR "Insights" OR "Statistics" OR "Business Analy*" OR "business intelligence")Limits applied](https://www.proquest.com/recentsearches.recentsearchtabview.recentsearchesgridview.scrolledrecentsearchlist.checkdbssearchlink:rerunsearch/8EA2D9A100774048PQ/None?site=pqdtglobal&t:ac=RecentSearches) |
| --- | --- |
|  | Databases: |
|  | ProQuest Dissertations & Theses Global |
|  | Limited by: |
|  | Date: From 01 January 2019 to 31 December 2022 |
|  | Language: |
|  | English |
|  |  |
|  |  |
| **S13** | [ab(NHS OR "National Health Service" OR "Public Health England" OR "Department of Health" OR "English healthcare" OR "English Health Care")Limits applied](https://www.proquest.com/recentsearches.recentsearchtabview.recentsearchesgridview.scrolledrecentsearchlist.checkdbssearchlink:rerunsearch/7AC3415585C84118PQ/None?site=pqdtglobal&t:ac=RecentSearches) |
|  | Databases: |
|  | ProQuest Dissertations & Theses Global |
|  | Limited by: |
|  | Date: From 01 January 2019 to 31 December 2022 |
|  | Language: |
|  | English |
|  |  |
|  |  |
| **S14** | [ab("competitive tendering" OR fundholding OR fund-holding OR "strategic contracting" OR "strategic buying" OR procurement OR purchas* OR "contracting out" OR gpfh OR "Primary Care Groups" OR "PCG*" OR "Primary Care Trust*" OR "PCT*" OR "Clinical Commissioning Group*" OR "CCG*" OR "commissioning" OR commissioner* OR "Assessing needs" OR "reviewing service provisions" OR "deciding priorities" OR "designing services" OR "shaping structure of supply" OR "planning capacity and managing demand" OR "supporting patient choice" OR "managing performance" OR "seeking public and patient views" OR "Needs assessment" OR "planning" OR "Market management" OR "Market development" OR decommissioning OR disinvestment OR "performance management" OR "procuring services" OR "monitoring and evaluation")Limits applied](https://www.proquest.com/recentsearches.recentsearchtabview.recentsearchesgridview.scrolledrecentsearchlist.checkdbssearchlink:rerunsearch/F3E3AC43FEA34199PQ/None?site=pqdtglobal&t:ac=RecentSearches) |
|  | Databases: |
|  | ProQuest Dissertations & Theses Global |
|  | Limited by: |
|  | Date: From 01 January 2019 to 31 December 2022 |
|  | Language: |
|  | English |
|  |  |
|  |  |
| **S15** | [ab("Primary Care" OR "Primary healthcare" OR "Primary Health Care" OR "Primary Health-Care" OR "Primary Health" OR phc OR "general practi*")Limits applied](https://www.proquest.com/recentsearches.recentsearchtabview.recentsearchesgridview.scrolledrecentsearchlist.checkdbssearchlink:rerunsearch/FDCBBF6083A743EBPQ/None?site=pqdtglobal&t:ac=RecentSearches) |
|  | Databases: |
|  | ProQuest Dissertations & Theses Global |
|  | Limited by: |
|  | Date: From 01 January 2019 to 31 December 2022 |
|  | Language: |
|  | English |
|  |  |
|  |  |
|  |  |
|  |  |
| **S16** | [s15 AND s14 AND s13 AND s12](https://www.proquest.com/recentsearches.recentsearchtabview.recentsearchesgridview.scrolledrecentsearchlist.checkdbssearchlink:rerunsearch/95426801421449DCPQ/None?site=pqdtglobal&t:ac=RecentSearches) |
|  | Databases: |
|  | ProQuest Dissertations & Theses Global |
