# Supplementary material for: The usage of data in NHS primary care commissioning: a realist review
Source: BMC Med. 2023 Jul 3;21:236. doi: 10.1186/s12916-023-02949-w (PMC10318817; doi:10.1186/s12916-023-02949-w)
Supplement: Supplementary file 4 — Additional file 4. Table of included studies. [file 12916_2023_2949_MOESM4_ESM.docx]

| **Study name** | **Year published** | **Authors (note: authors are shown in the order listed on article)** | **Objectives** | **Setting/methods** | **Type of paper** | **Type of study** |
| --- | --- | --- | --- | --- | --- | --- |
| (Re)Surveying the Uses of Geographical Information Systems in Health Authorities 1991-2001 | 2003 | Smith, D.P.; Gould, M.I.; Higgs, G. | To provide a comparative analysis of two separate national surveys of the uptake and use of Geographical Information Systems (GIS) by Health Authorities (HAs) in England and Wales | Cummins and Rathwell (1991) undertook an early study of the use of GIS by health organisations, on behalf of the Association of Geographical Information (AGI). A postal questionnaire was distributed amongst Information Technology/Services personnel working in the 104 HAs in England and Wales that were in existence during Summer 2001. This paper compares the 1991 survey results with the 2001 updated survey data. | Journal article, peer-reviewed research | Survey analysis |
| A matter of facts | 2008 | Dewhurst, R. | Opinion piece on world-class commissioning and information in the NHS | N/a - opinion piece | Opinion piece | Grey literature (opinion piece) |
| Accountable to whom, for what? An exploration of the early development of Clinical Commissioning Groups in the English NHS | 2013 | Checkland K.; Allen P.; Coleman A.; et al | To explore CCG's developing accountability relationships. | The researchers carried out detailed case studies in eight CCGs, using interviews, observation, and documentary analysis to explore their multiple accountabilities. | Journal article, peer-reviewed research | Qualitative research (case studies) |
| Addiction to Medicine | 2010 | The National Treatment Agency for Substance Misuse | To provide: -An analysis of relevant National Drug Treatment Monitoring System (NDTMS) data and prescription data to investigate prevalence and trends -Structured interviews with targeted PCTs/partnerships to better understand the commissioning, governance (of prescribing and drug treatment provision) and provision of drug treatment services -To complete Surveys and structured interviews with specialist drug treatment providers and dedicated providers of treatment for POM/OTC medicines dependency to determine what is being provided and how local services are configured | This report considers the problems encountered by the use of psychotropic and opioid analgesic medication (such as diazepam and tramadol) in the general population, (including that encountered by people reporting problems with illegal drugs) and the role of health services, including specialist addiction, pain and primary care services in order to address these issues. | NHS report (published by the National Treatment Agency for Substance Misuse) | Grey literature (mixed methods) |
| An exploratory study of the role and training needs of one primary care trust’s professional executive committee members | 2005 | Offredy, M. | This paper provides a summary of an exploratory study based on an interpretive approach, aiming to elucidate how professional executive committee members of one primary care trust see their role and the training they need to fulfil their functions. | Using questionnaires and semi-structured interviews, the study examined the perceived training needs of the 26 participants. | Journal article, peer-reviewed research | Qualitative research (interviews) |
| An integrated treatment algorithm for pharmacotherapy and psychotherapy | 1999 | Kisely, S. R.; Jones J. | This paper describes how Birmingham Health Authority (HA), the largest HA in England and Wales with a population of just under one million, developed and implemented an evidence-based strategy for the use of psychotherapy services. | A literature search and health needs assessment for psychotherapy in Birmingham was performed. | Journal article, peer-reviewed research | Literature review |
| Building high-quality commissioning : what role can external organisations play? | 2010 | Naylor, C.; Goodwin N. | The primary aim of this research was to examine how, in the context of world class commissioning, external support is being used by PCTs and SHAs and whether it is helping to develop more effective commissioning. | The study was based primarily on interviews and focus groups with a range of stakeholders, as well as online surveys. | Grey literature (King's Fund report) | Grey literature (mixed methods) |
| Can general practice data be used for needs assessment and health care planning in an inner-London district? | 1995 | Scobie, S.; Basnett., I; McCartney, P. | To evaluate the quality of information in six computerized GP practices. | A comparison was carried out of the recording of registration and social information, health risk factors, medication and record on consultations on the computer and in the manual records for a sample of patients, with an audit of morbidity coding by computer. A comparison was made of computerised disease registers with prescribing for diseases. | Journal article, peer-reviewed research | Evaluation |
| Care home services at the vanguard: a qualitative study exploring stakeholder views on the development and evaluation of novel, integrated approaches to enhancing healthcare in care homes | 2018 | Stocker R.; Bamford, C.; Brittain, K.; et al | To explore stakeholders’ understanding of novel integrated approaches to enhancing care in care homes (a care home ‘vanguard’) and identify priorities for evaluation. | Qualitative study, using semistructured interviews with commissioners and service providers to/within care homes, and third sector organisations with thematic analysis in a CCG area in England | Journal article, peer-reviewed research | Qualitative research (interviews) |
| CCGs vary widely in how well they deliver healthcare, new measure shows | 2016 | Hawkes, N. | To analyse and describe how new indicators show that primary care clinical commissioning groups (CCGs) in England vary widely in their ability to deliver healthcare equitably to rich and poor people. | N/a - descriptive/opinion piece | Journal article, peer-reviewed research | Descriptive news article |
| Challenges to using evidence from systematic reviews to stop ineffective practice: an interview study | 2013 | Shepperd, S.; Adams, R.; Hill, A.; et al | To examine the challenges to using systematic review evidence to develop guidance for decommissioning ineffective health services, and the problems experienced by clinicians and commissioners when they attempt to implement the evidence from this guidance. | Completed interviews with 23 clinicians and 15 commissioners from nine commissioning organizations (Primary Care Trusts) in the south of England. | Journal article, peer-reviewed research | Qualitative research (interviews) |
| Clinical commissioning groups: Supporting improvement in general practice? | 2013 | Naylor, C.; Curry, N.; Holder, H.; et al | This report is based on fieldwork conducted before CCGs had become fully authorised. It provides an overview of developments through to March 2013, plus an assessment of the opportunities and challenges ahead. | The research is based on 74 in-depth interviews, 18 direct observations and a survey of all general practices in our case study sites, to which there were 232 responses. | Grey literature (King's Fund report) | Grey literature (mixed methods) |
| Clinical governance, education and learning to manage health information | 2011 | Ellis B.; Howard, J. | This paper aims to suggest that the concept of clinical governance goes beyond a bureaucratic accountability structure and can be viewed as a negotiated balance between imperfectly aligned and sometimes conflicting goals within a complex adaptive system. | The study, located within the English National Health Service (NHS) between 1999 and 2005, is case study based using a multi method approach to data collection within two primary care organisations (PCOs). | Journal article, peer-reviewed research | Qualitative research (case studies) |
| Clinical leadership in service redesign using Clinical Commissioning Groups: a mixed-methods study | 2018 | Storey, J.; Holti, R.; Hartley, J.; et al | This research examined the extent to which, and the methods by which, clinicians stepped forward to take up a leadership role in service redesign using CCGs as a platform. | The project proceeded in five phases: (1) a scoping study across 15 CCGs, (2) the design and administration of a national survey of all members of CCG governing bodies in 2014, (3) six main in-depth case studies, (4) a second national survey of governing body members in 2016, which allowed longitudinal comparisons, and (5) international comparisons. | Report (NIHR) | Mixed methods research |
| Commissioning and funding general practice: Making the case for family care networks | 2014 | Addicott, R.; Ham, C. | This paper describes the current system of commissioning and funding general practice and how this is being used in four areas of England to develop innovative models of primary care provision. | See objectives | Grey literature (King's Fund report) | Grey literature (opinion piece) |
| Commissioning for equity in the NHS: rhetoric and practice | 2015 | Wenzl, M.; McCuskee, S.; Mossialos, E. | To review evidence on equity as a policy goal of resource allocation in the English NHS, focussing on the role of CCGs and their capacity to achieve equity through the process of commissioning | Systematic review | Journal article, peer-reviewed research | Systematic review |
| Commissioning healthcare for people with long term conditions: the persistence of relational contracting in England’s NHS quasi-market | 2013 | Porter, A.; Mays, N.; Shaw, S.E.. et al. | To examine the extent to which local commissioners have adopted a market-oriented (transactional) model of commissioning of care for people with long term conditions several years into the latest period of market-oriented reform. | The authors studied the commissioning of services for people with three long term conditions - diabetes, stroke and dementia - in three English primary care trust (PCT) areas over two years (2010-12). They took a broadly ethnographic approach to understanding the day-to-day practice of commissioning. | Journal article, peer-reviewed research | Multimethod qualitative research |
| Commissioning high-quality care for people with long-term conditions | 2013 | Smith JA; Shaw S; Porter A; et al | This study explored what commissioners actually do to commission care for people with long-term conditions, and how this might be improved. | The research is based on 15 months of detailed observation from November 2010 to January 2012 in three commissioning communities: Calderdale, Somerset and the Wirral. | Grey literature (Nuffield Trust) | Grey literature (qualitative) |
| Commissioning in the reformed NHS: policy into practice | 2006 | Wade, E; Smith, J.A.; Peck, E.; et al | This report focuses on a detailed analysis of what the commissioning function post Commissioning a Patient-Led NHS will actually entail – the tasks and activities that will need to be carried out by PCTs or their agents. | See objectives | University research report | Grey literature (policy paper) |
| Commissioning NHS dentistry in England: Issues for decision-makers managing the new contract with ﬁnite resources | 2009 | Holmes, R. D.; Bate, A.; Steele, J. G.; et al | To explore the views of dental decision-makers in Primary Care Organisations with regard to the management of NHS dental services, and to gauge participants' awareness of economics-based approaches including programme budgeting and marginal analysis, with which to potentially structure commissioning decisions. | Recorded semi-structured interviews were conducted with 18 NHS dental decision-makers (mixed clinical and finance backgrounds) predominantly across Primary Care Trusts in England. | Journal article, peer-reviewed research | Qualitative research (interviews) |
| Commissioning of self-management support for people with long-term conditions: an exploration of commissioning aspirations and processes | 2016 | Reidy C; Kennedy A; Pope C; et al | To explore how self-management support (SMS) is considered and conceptualised by Clinical Commissioning Groups (CCGs) and whether this is reflected in strategic planning and commissioning. | Consisted of a multisite, quasi-ethnographic exploration of 9 CCGs. | Journal article, peer-reviewed research | Qualitative research (ethnography) |
| Commissioning through Competition and Cooperation: final report | 2016 | Allen P; Osipovic, D; Shepherd, E; et al | This project aimed to investigate how commissioners in local health systems managed the interplay of competition and cooperation in their local health economies, looking at acute and community health services (CHS). | The project consisted of four in depth case studies of four local health economies located in four CCG areas across England. | Report | Grey literature (case studies) |
| Cost-effectiveness analysis and formulary decision making in England: Findings from research | 2007 | Williams, I; Bryan, S | This paper reports research on the use of economic evaluations in technology coverage decisions in England. | There were two strands to the research. The first involved collation and analysis of information proformas used by decision-making committees when considering proposed new technologies—referred to hereafter as New Technology Request Forms (NTRFs). The second strand involved qualitative case studies of decision-making committees. | Journal article, peer-reviewed research | Mixed methods research |
| Creativity and service innovation: an examination of differences between theory and practice | 2016 | Gordon, S | This study addresses creativity and innovation literatures and explores the necessity for creativity in the implementation of service innovations in the English National Health Service. | Based on a qualitative research design, and using a critical realist approach, this research unearths a rich seam of empirical data through observations and semi-structured interviews in an English National Health Service primary care organisation, known as a NHS Clinical Commissioning Group | PhD Thesis | PhD thesis |
| Designing whole-systems commissioning: Lessons from the English experience | 2013 | Williams, I; Bovaird, T; Brown, H; et al | The primary aims of this study were to identify determinants of successful strategic commissioning and to assess the overall state of current knowledge. | A review of the published literature and interviews with those involved in strategic commissioning in England. | Journal article, peer-reviewed research | Multimethod (literature review and qualitative data) |
| Determining priorities for change in primary care: the value of practice-based needs assessment | 1997 | Ruta, D. A.; Duffy, M. C.; Farquharson, A; et al | To examine whether information on health and health care needs, when used as the basis for a priority setting exercise, can provide a useful first step in planning primary care provision within a practice. | A three-stage process of information-gathering from a number of sources, including continuous data recording of patient contacts and a postal survey of all adults registered with the practice, identification of key findings and discussion of associated issues, and priority setting of proposals for practice development using the nominal group technique. | Journal article, peer-reviewed research | Mixed methods research |
| Developing a prioritisation framework in an English Primary Care Trust | 2006 | Wilson, E; Rees, J; Fordham, R | The researchers developed a weighted benefit score framework for use in an English PCT which ranked options in order of 'cost-value' or 'cost per point of benefit'. | Development of a weighted benefit score framework for use in an English PCT | Journal article, peer-reviewed research | Feasibility study (development of a tool to help commissioners) |
| Developing the infrastructure to support the optimisation of antibiotic prescribing using the learning healthcare system to improve healthcare services in the provision of primary care in England | 2020 | Palin, V; Tempest, E; Mistry, C; et al | The learning healthcare system (LHS) underpinned by data analysis and feedback to clinical care providers is thought to improve quality of care. This work aimed to implement an LHS for antibiotic prescribing in primary care in England. | Deidentified patient-level data from general practices were processed and analysed at regular intervals (fortnightly increments). A dashboard application was developed and implemented displaying analytical graphics to give periodic feedback to clinicians, tailored to each clinical site. Benchmarking parameters were established by the analysis of two large national primary care datasets allowing peer-to-peer comparisons. | Journal article, peer-reviewed research | Feasibility study (development of a tool to help commissioners) |
| Does access to a demand-led evidence briefing service improve uptake and use of research evidence by health service commissioners? A controlled before and after study | 2017 | Wilson, P.M.; Farley, K; Bickerdike, L; et al. | The researchers undertook a controlled before and after study to evaluate whether access to a demand-led evidence briefing service improved the use of research evidence by commissioners compared with less intensive and less targeted alternatives. | Nine Clinical Commissioning Groups (CCGs) in the North of England received one of three interventions: (A) access to an evidence briefing service; (B) contact plus an unsolicited push of non-tailored evidence; or (C) unsolicited push of non-tailored evidence. | Journal article, peer-reviewed research | Controlled before and after study |
| English national health service's savings plan may have helped reduce the use of three 'low-value' procedures | 2015 | Coronini-Cronberg, S; Bixby, H; Laverty, A. A.; et al | The National Health Service (NHS) must seek reductions in health expenditures of 17 percent over four years. The spending cuts were to be achieved through improvements in service quality and efficiency, including reducing the use of ineffective, overused, or inappropriate procedures. However, the NHS left it to the local commissioning (or funding) organizations, known as primary care trusts, to determine what steps to take to reduce spending. | To assess whether the initiative had an impact, we examined six low-value procedures: spinal surgery for lower back pain, myringotomy to relieve eardrum pressure, inguinal hernia repair, cataract removal, primary hip replacement, and hysterectomy for heavy menstrual bleeding. | Journal article, peer-reviewed research | Evaluation |
| Evaluating the design and implementation of the whole systems integrated care programme in North West London: why commissioning proved (again) to be the weakest link | 2019 | Smith, J; Wistow, G; Holder, H; et al. | The authors examined the role of commissioning in attempts to secure large-scale change within and between health and social care services to support the delivery of integrated care for people living with complex long-term conditions. | The authors analysed data collected in semi-structured interviews, surveys, workshops and non-participant observations using a thematic framework derived both deductively from the literature on commissioning and integrated care | Journal article, peer-reviewed research | Multimethod qualitative research |
| Evidence based policy making and the ‘art’ of commissioning – how English healthcare commissioners access and use information and academic research in ‘real life’ decision-making: an empirical qualitative study | 2015 | Wye, L; Brangan, E; Cameron, A; et al | Policymakers such as English healthcare commissioners are encouraged to adopt ‘evidence-based policy-making’, with ‘evidence’ defined by researchers as academic research. To learn how academic research can influence policy, researchers need to know more about commissioning, commissioners’ information seeking behaviour and the role of research in their decisions. | In case studies of four commissioning organisations, we interviewed 52 people including clinical and managerial commissioners, observed 14 commissioning meetings and collected documentation e.g. meeting minutes and reports. Using constant comparison, data were coded, summarised and analysed to facilitate cross case comparison. | Journal article, peer-reviewed research | Qualitative research (case studies) |
| Evidence in management decisions (EMD) _advancing knowledge utilization in healthcare management | 2012 | Swan, J.; Clarke, A.; Nicolini, D.; et al | To investigate the utilisation of evidence in actual healthcare commissioning decisions | Detailed case studies focusing on commissioning practices in 4 NHS commissioning organisations (PCTs), which were chosen to capture variation in context. A nationally representative survey of individuals involved in commissioning. | NIHR Report | Qualitative research (case studies) |
| Evidence use in decision-making on introducing innovations: a systematic scoping review with stakeholder feedback | 2017 | Turner, S.; D’Lima, D.; Hudson, E.; et al. | To understand how decision-making processes at the professional group, organisational, and local system level influence the use of evidence in decisions to adopt innovations in acute and primary health care? | A systematic scoping review was used to review the health literature on innovations within acute and primary care and map processes at the professional, organisational and local system levels which influence how evidence informs decision-making on innovation. | Journal article, peer-reviewed research | Systematic review |
| Evidence-based commissioning in the English NHS: who uses which sources of evidence? A survey 2010/2011 | 2013 | Clarke, A.; Taylor-Phillips, S.; Swan, J.; et al | To investigate types of evidence used by healthcare commissioners when making decisions and whether decisions were influenced by commissioners’ experience, personal characteristics or role at work. | Cross-sectional survey of 345 National Health Service (NHS) staff members. The study was conducted across 11 English Primary Care Trusts between 2010 and 2011. | Journal article, peer-reviewed research | Survey analysis |
| Evidence-based policy-making in the NHS: exploring the interface between research and the commissioning process. | 1999 | Harries, U.; Elliott, H.; Higgins, A. | To identify factors which facilitate or impede evidence-based policy-making at a local level in the NHS. | The study involved a literature review and case studies of social research projects which were initiated by NHS health authority managers or general practitioner (GP) fundholders in one region of the NHS. Data were collected through in-depth interviews with lead policy-makers, GPs and researchers working on each of the case studies and analysis of project documentation. | Journal article, peer-reviewed research | Multimethod (literature review and qualitative data) |
| Exploring the early workings and impact of emerging Clinical Commissioning Groups: Final report | 2012 | Checkland, K.; Coleman, A.; McDermott, I.; et al | The aim of the study was to explore the early experiences of emerging Clinical Commissioning Groups as they set themselves up as ‘Pathfinders’ and moved towards authorisation, investigating the factors that had affected their development and drawing out lessons for the future | Case studies in eight CCGs, along with national web surveys at two points in time and telephone interviews with a random sample of CCGs. | Report (published by the Policy Research Unit in Commissioning and the Healthcare System Manchester Centre for Health Economics) | Grey literature (case studies) |
| Exploring the GP ‘added value’ in commissioning: What works, in what circumstances, and how? | 2015 | Mcdermott, I.; Coleman, A.; Perkins, N.; et al | This report presents the findings from a second phase of our ongoing study following the development of CCGs in England since 2011. The aim of the second phase of our study was therefore to follow up those claims made in the first phase around issues of GP ‘added value’. We explored further the potential added value that clinicians, specifically GPs, bring to the commissioning process in interviews, and followed this up with observations of commissioners at work. | Using semi-structured interviews with clinicians and managers, observations of a range of CCG meetings in 4 sites | Report (published by the Policy Research Unit in Commissioning and the Healthcare System Manchester Centre for Health Economics) | Grey literature (multimethod qualitative) |
| Exploring the ongoing development and impact of Clinical Commissioning Groups | 2014 | Checkland, K.; Coleman, A.; Perkins, N.; et al | To explore the impacts of CCGs, with a particular focus upon the potential added value that clinicians bring to the commissioning process (in its widest sense), and to elucidate the contexts and factors that enable or inhibit the delivery of these benefits. | The findings presented here represent the second stage of a longitudinal project tracking the development and early activities of CCGs. For this second stage of the research we are tracking the ongoing development of the case study CCGs | Report (published by the Policy Research Unit in Commissioning and the Healthcare System Manchester Centre for Health Economics) | Grey literature (case studies) |
| Facilitating large-scale implementation of evidence based health care: insider accounts from a co-operative inquiry | 2015 | Waterman, H.; Boaden, R.; Burey, L.; et al. | To understand how knowledge transfer associates (KTAs) working as part of the UK National Institute for Health Research ‘Collaboration for Leadership in Applied Health Research and Care’ for Greater Manchester (GM CLAHRC) facilitated the implementation of EBHC across several commissioning and provider health care agencies. | A prospective co-operative inquiry with eight Knowledge Transfer Associates (KTAs) was carried out comprising of 11 regular group meetings where they reflected critically on their experiences. Twenty interviews were also conducted with other members of the GM CLAHRC Implementation Team to gain their perspectives of the KTAs facilitation role and process. | Journal article, peer-reviewed research | Evaluation |
| Facing the Future: A Study of the Changing Pattern of Contracting within a National Health Service Community Trust 1994-2001 | 2002 | Cowie, A | This study examines the policy formation processes involved in contracting for community health care services between 1994 and 2001. | Interviews and non-participant observations of key meetings and seminars, supported by documentary evidence from both published material in the public domain and internal documents from stakeholder organisations. | PhD Thesis | PhD thesis |
| From data to decisions? Exploring how healthcare payers respond to the NHS Atlas of Variation in Healthcare in England | 2014 | Schang, L.; Morton, A.; DaSilva, P.; et al | To explore to what extent and how Primary Care Trusts (PCTs) in England have used the NHS Atlas of Variation in Healthcare | Data collection involved an email survey among PCT Chief Executives and a telephone follow-up to reach non-respondents (total response: 53 of 151 of PCTs, 35%). 45 senior to mid-level staff were interviewed to probe themes emerging from the survey. The data were analysed using a matrix-based Framework approach. | Journal article, peer-reviewed research | Mixed methods research |
| GP Champions for Youth Health: Commissioning effective primary care services for young people | N/a | Association for Young People’s Health (AYPH), the Royal College of General Practitioners’ (RCGP) Adolescent Health Group and Youth Access. | Briefing aimed at policymakers, commissioners and funders, including independent funders with an interest in health and health-related services for young people up to 25 years. Its purpose is to support improvements in young people’s access to Primary Care services | Briefing paper bringing together some of the lessons learnt during the GP Champions project. It is intended to help policymakers and commissioners think about steps they can take to secure a better investment in young people’s health. | Briefing paper for policymakers | Briefing paper |
| How are policy makers using evidence? Models of research utilisation and local NHS policy making | 2000 | Elliott, H.; Popay, J. | To identify factors that facilitate or impede evidence-based policy making at a local level in the UK National Health Service (NHS). | One NHS region in England. | Journal article, peer-reviewed research | Multimethod (literature review and qualitative data) |
| Implementing world class commissioning competencies | 2012 | McCafferty, S.; Williams, I.; Hunter, D.; et al | To explore the development and implementation of world class commissioning and draws implications for future commissioning arrangements. | This research draws on interviews with key informants (n = 6) and a literature review to analyse the aims of, and stimulus for, WCC. In-depth interviews (n = 38) were conducted in three PCTs in the north of England in 2009 to analyse the interpretation and implementation of WCC. | Journal article, peer-reviewed research | Multimethod (literature review and qualitative data) |
| Improving the capabilities of NHS organisations to use evidence: a qualitative study of redesign projects in Clinical Commissioning Groups | 2017 | Swan, J.; Gkeredakis, E.; Manning, RM.; et al | To understand practices and conditions (which we term ‘capabilities’) that enable evidence use in commissioning work. We consider how research gets into CCGs (‘push’), how CCGs use evidence (‘pull’) and how this can be supported (toolkit development). | Semistructured ethnographic interviews in eight CCGs. We also conducted observations of redesign meetings in two of the CCGs. | NIHR Report | Grey literature (multimethod qualitative) |
| Informing Healthier Choices: Information and Intelligence for Healthy Populations | Unknown | Department of Health | Strategy piece - the document sets out proposals to provide comprehensive and innovative information systems that can make a real difference to delivery of these plans in a cost effective fashion. The ultimate aim is health improvement but these proposals would also underpin evidence based commissioning of services, as well as providing more precise and meaningful monitoring of performance of public service activities. | See objectives | Government report | Grey literature (strategy piece) |
| Introducing care pathway commissioning to primary dental care: measuring performance | 2011 | Harris, R.; Bridgman, C.; Ahmad, M.; et al | To describe data from a pilot use of a blended contract using key performance indicators related to primary dental care | To report on the findings of a project involving three dental practices implementing a system based on rating patients according to their risk of disease and need for care are outlined by reporting data from surgery-based clinical databases and interviews from commissioners and providers | Journal article, peer-reviewed research | Mixed methods research |
| Investigating recent developments in the commissioning system | 2018 | Moran, V.; Allen, P.; McDermott, I. | The questions addressed by the research were: 1. How are CCG internal processes of decision making changing? 2. What is the role of the individual CCG in the current commissioning landscape? 3. How is accountability maintained by CCGs in the current commissioning landscape? 4. How is competition and the current pricing regime relevant to CCGs’ commissioning decisions? 5. How should commissioning develop? | The design of the study consisted of three case study sites centred round three CCG areas, spread across England. Our main method of data collection was interviews with senior commissioners in the CCGs and senior managers in a selection of local provider organisations and local authorities. We collected additional data by examining locally produced documents, such as STP plans | Report (PRU Comm) by PRU Comm, a collaboration between London School of Hygiene and Tropical Medicine, the University of Manchester, the Centre for Health Services Studies at the University of Kent and the Personal Social Services Research Unit. | Grey literature (case studies) |
| Investigating the process of community profile compilation | 1996 | Billings, J. | To provide a critical review of the data collection processes involved in the compilation of a community profile for one area within the context of the NHS reforms | To investigate the data collection processes involved in compiling a community profile to assess family health needs from the perspective of one fund-holding GP. | Journal article, peer-reviewed research | Evaluation |
| Knowledge exchange in health-care commissioning: case studies of the use of commercial, not-for-profit and public sector agencies, 2011–14 | 2015 | Wye, L; Brangan, E; Cameron, A; et al | To study knowledge exchange between these external providers and health-care commissioners to learn about knowledge acquisition and transformation, the role of external providers and the benefits of contracts between external providers and health-care commissioners. | Using a case study design, we collected data from eight cases, where commercial and not-for-profit organisations were contracted. We conducted 92 interviews with external providers (n = 36), their clients (n = 47) and others (n = 9), observed 25 training events and meetings and collected various documentation including meeting minutes, reports and websites. | NIHR Report | Grey literature (case studies) |
| Leadership of healthcare commissioning networks in England: a mixed-methods study on clinical commissioning groups | 2013 | Zachariadis, M; Oborn, E; Barrett, M; et al. | To explore the relational challenges for general practitioner (GP) leaders setting up new network-centric commissioning organisations in the recent health policy reform in England | Mixed-method, multisite and case study research in six clinical commissioning groups and local clusters in the East of England area | Journal article, peer-reviewed research | Qualitative research (case studies) |
| LSE/ Right Care project on NHS Commissioners’ use of the NHS Atlas of Variation in Healthcare: Case studies of local uptake | 2012 | Schang, L.; Morton, A. | To understand how the Right Care Atlas series is being used within PCT clusters and CCGs to inform planning activities, and identify levers and barriers to use | Case studies of several NHS organisations | Report | Grey literature (case studies) |
| National evaluation of general practitioner commissioning pilots: lessons for primary care groups | 2000 | Smith. J; Regen, E; Shapiro, J; et al | To monitor the development of the 40 national pilot sites, identify the factors that inhibited or facilitated progress, and consider the implications for the implementation and development of primary care groups (PCGs). | Semi-structured face-to-face interviews with GPs, health authority (HA) managers, and pilot managers from each of the 40 pilot sites (141 interviews in total) and focus group discussions with nurses, social services officers, and community health council officers in the 40 sites. | Journal article, peer-reviewed research | Multimethod qualitative research |
| Negotiating commissioning pathways for the successful implementation of innovative health technology in primary care | 2019 | Maniatopoulos, G.; Haining, S.; Allen, J. | To explore the process by which commissioning organisations make their decisions to commission innovative health technologies. | Case study of four Clinical Commissioning Groups (CCGs) involved in the commissioning of services in primary and secondary care. Semi-structured in-depth interviews (16 in total) and two focus groups | Journal article, peer-reviewed research | Qualitative research (case studies) |
| NHS commissioning practice and health system governance: a mixed-methods realistic evaluation | 2015 | Sheaff, R.; Charles, N.; Mahon, A.; et al | To: elicit the programme theory of NHS commissioning policy and empirically test its assumptions; explain what shaped NHS commissioning structures; examine how far current commissioning practice allowed commissioners to exercise governance over providers; examine how commissioning practices differ in different types of commissioning organisation and for specific care groups; and explain what factors influenced commissioning practice and the relationships between commissioners and providers. | Mixed-methods realistic evaluation with four English case study sites (CCGs) | NIHR publication | Grey literature (case studies) |
| Perceptions of the uses of routine general practice data beyond individual care in England: a qualitative study | 2018 | Wyatt, D.; Cook, J.; McKevitt, C | To investigate how different lay and professional groups perceive and understand the use of routinely collected general practice patient data for research, public health, service evaluation and commissioning. | Multimethod, qualitative study including participant observation of the design and delivery of a series of deliberative engagement events about a local patient database made of routine primary care data. The researchers also completed semistructured interviews with key professionals involved in the database. The research took place in an inner city borough in England. | Journal article, peer-reviewed research | Multimethod qualitative research |
| Power to the people | 2009 | Shepherd, S. | To describe how shared data is providing a critical tool through which NHS services can improve their understanding of their local populations. | Analysis piece in journal supplement, includes two examples of goof practice of data sharing | Journal supplement | Descriptive piece |
| Practice makes perfect: “GP-led commissioning will only succeed if the new consortia learn the lessons of NHS Cumbria" | 2010 | Corrigan, P | To argue that creating general practitioners (GP) commissioning consortia will only take place if time and effort are given to the development of GPs as leaders and members of these consortia | Pamphlet published by a think tank based around a single case study in Cumbria. | Think tank (Demos) publication | Grey literature (think tank piece) |
| Practice-based Commissioning: Reinvigorate, Replace or Abandon? | 2008 | Curry, N.; Goodwin, N.; Naylor, C.; et al | To assess the progress made in meeting three objectives (encouraging clinical engagement in service redesign and development, bringing about better, more convenient, services for patients, and enabling the better use of resources) related to Practice Based Commissioning | In-depth interviews conducted with a range of key stakeholders in four PCT sites | Grey literature (King's Fund) | Grey literature (interviews) |
| Prioritising public health: a qualitative study of decision making to reduce health inequalities | 2011 | Orton, LC.; Lloyd-Williams, F.; Taylor-Robinson, DC.; et al | To explore the current experiences of those involved in decision making to reduce health inequalities with cardiovascular disease (CVD) as a case study. | In-depth qualitative study employing 40 semi-structured interviews and three focus group discussions. Participants were public health policy makers and planners in CVD in the UK, including: Primary Care Trust and Local Authority staff (in various roles); General Practice commissioners; public health academics; consultant cardiologists; national guideline managers; members of guideline development groups, civil servants; and CVD third sector staff. | Journal article, peer-reviewed research | Multimethod qualitative research |
| Prioritizing investment in public health and health equity: what can commissioners do? | 2013 | Marks, L.; Weatherly, H.; Mason, A. | To explore commissioners' views on prioritizing for investment in health | Decision makers' views were sought through semi-structured interviews and an online survey, and prioritization tools were reviewed. Interviews were held in 2008-2009 with a subsample followed up in 2009-2010. In late 2009, a national online survey was sent to 508 individuals across 146 primary care trusts (PCTs). | Journal article, peer-reviewed research | Mixed methods research |
| Priority Setting among Primary Care Trusts in Northwest England: Approaches, Processes and Use of Evidence | 2007 | Chinamasa, C. | To explore the approaches, processes and use and presentation of evidence for setting priorities among PCTs within a Strategic Health Authority in the North West of England. | Data were collected using in-depth semi-structured interviews among samples of people involved in setting priorities at PCTs A (9 out of the 11 people identified) and B (13 out of the 25 identified); a self-administered postal questionnaire survey among 85 (out of 170) people involved in Tier 2 priority setting at 11 (out of 14) PCTs within Greater Manchester and a focus group discussion among members of the Professional Executive Committee (PEC) at PCT C. | Grey literature (PhD thesis) | PhD thesis |
| Reconciling putting people first and world class commissioning: A case study | 2009 | Klee, D. | To describe the challenges faced by a Council and PCT in understanding and agreeing shared priorities and how they used this whole‐systems framework to keep a clear focus on what local people want and need | Descriptive piece by author based on their experience | Feature piece in journal | Descriptive piece |
| Requisite models for strategic commissioning: the example of type 1 diabetes | 2008 | Airoldi, M.; Bevan, G.; Morton, A.; et al | To develop a framework for cost-effectiveness analysis to provide information for organisations responsible for strategic commissioning of health services for defined populations and illustrates its use by modelling intensive glucose control in type 1 diabetes in England. | This paper outlines a framework that estimates the health benefits and costs of different interventions and shows how this requires modelling to produce estimates in a way that is transparent to commissioners, of requisite complexity to produce sound estimates for priority setting using routinely available data. | Journal article, peer-reviewed research | Framework development |
| Rhetoric, evidence and policymaking: a case study of priority setting in primary care | 2009 | Russell, J.; Greenhalgh, T. | To contribute to scholarly inquiry about how policymakers talk about and reason with evidence by exploring the microprocesses of deliberation and specifically the meaning-making practices of a group of people charged with prioritising health care within an NHS Primary Care Trust in the UK. | Case study of a Priorities Forum of an NHS Primary Care Trust (PCT) based on meeting observations and interviews | Final report of a research project | Grey literature (case studies) |
| Risk or reward? The Changing Role of CCGs in General Practice | 2015 | Holder, H.; Robertson, R.; Ross, S.; et al | To understand the development of CCGs, and to understand how CCGs are functioning as membership organisations and how they are supporting the development of primary care in their local area. | Report based on a survey, interviews, observations and reviews of board papers | Grey literature (King's Fund) | Grey literature (mixed methods) |
| Structures and processes for priority-setting by health-care funders: a national survey of primary care trusts in England | 2012 | Robinson, S.; Dickinson, H.; Freeman, T.; et al | To undertake a survey of local resource allocators in the English National Health Services in order to map and explore current priority-setting activity. | A national survey was sent to Directors of Commissioning in English Primary Care Trusts (PCTs) and analysed | Journal article, peer-reviewed research | Survey analysis |
| The capacity of health service commissioners to use evidence: a case study | 2018 | Currie, G.; Croft, C.; Chen, Y.; et al | To explore the capacity of CCG-led commissioning networks to make decisions that are based on evidence. | Study of 13 cases of representative (region, size, urban/rural) commissioning networks in England, drawing on interviews with commissioning managers, general practitioners (GPs), patient and public involvement (PPI) representatives and other relevant stakeholders | NIHR Report | Grey literature (interviews) |
| The effects of an evidence- and theory-informed feedback intervention on opioid prescribing for non-cancer pain in primary care: A controlled interrupted time series analysis | 2021 | Alderson SL.; Farragher, TM.; Willis, TA.; et al | To evaluate the effects of a comparative feedback intervention with persuasive messaging and action planning on opioid prescribing in primary care. | Interrupted time series analysis | Journal article, peer-reviewed research | Interrupted time series analysis |
| The impact of a quasi-market on sexually transmitted disease services in the UK | 1999 | Evans, D | To report the results of a study of the impact of quasi-market reforms on sexually transmitted disease (STD) services in one UK health region | A qualitative case-study approach to examine HA commissioning of STD services | Journal article, peer-reviewed research | Qualitative research (case studies) |
| The impact of the NHS market: An overview of the literature | 2010 | Brereton, L.; Vasoodaven, V. | To present the results of a literature search on the effectiveness of NHS ‘internal’ or ‘quasi’ market policies to promote competition among providers over the past 20 years. | Literature review | Grey literature (think tank report) | Grey literature (think tank piece) |
| The NHS deserves better use of hospital medicines data | 2020 | Goldacre B.; MacKenna, B. | To argue that that hospital medicines data has huge potential to improve patient care and that its use for public good is being blocked by cultural, contractual, and political barriers, rather than technical complexity | N/a - analysis piece | Journal article, peer-reviewed research | Opinion piece |
| The Use of External Consultants by NHS Commissioners in England: What Lessons Can Be Drawn for GP Commissioning? | 2011 | Naylor, C.; Goodwin, N. | To describe the current use of external support by commissioners in the English NHS, assess what impact it is having; explore the factors influencing effectiveness and draw out the implications for the future of NHS commissioning. | Two national surveys of primary care trust managers, qualitative interviews and focus groups with representatives of 12 firms providing support services to commissioners, focus groups in three primary care trusts and interviews with representatives of two strategic health authorities (SHAs). | Journal article, peer-reviewed research | Mixed methods research |
| The work of commissioning: a multisite case study of healthcare commissioning in England's NHS | 2013 | Shaw, S.; Smith, JA.; Porter, A.; et al | To examine the work of commissioning care for people with long-term conditions and the factors inhibiting or facilitating commissioners making service change. | Multisite mixed methods case study research, combining qualitative analysis of interviews, documents and observation of meetings across three 'commissioning communities' (areas covered by a primary care trust) in England | Journal article, peer-reviewed research | Mixed methods research |
| Together we are better? Strategic needs assessment as a tool to improve joint working in England | 2011 | Ellins, J.; Glasby, J. | To report the results of a national survey to explore preparations under way by local authorities and primary care trusts (PCT) for the duty to conduct a joint strategic needs assessment (JSNA), key barriers and enablers to this, and the implications for future policy | The researchers conducted a national survey of all PCT chief executives, directors of adult social services and public health in England (a total of 459 people). A qualitative survey was e‐mailed in February 2008 at the time when health and social care communities were preparing to implement the new duty. All completed surveys were analysed by both authors using thematic content analysis. | Journal article, peer-reviewed research | Survey analysis |
| Towards equitable commissioning for our multiethnic society: a mixed-methods qualitative investigation of evidence utilisation by strategic commissioners and public health managers | 2013 | Salway, S.; Turner, D.; Mir, G.; et al | To describe, across a range of commissioning contexts, how managers seek out, appraise and apply evidence relating to ethnic diversity and inequality alongside other forms of knowledge, and to identify factors (at evidence, individual and contextual levels and their interfaces) that support or inhibit the critical and effective use of evidence within the commissioning cycle and thereby identify promising routes of intervention. | In-depth semistructured interviews were conducted with 19 national key informants. Detailed case studies of real-world commissioning work were undertaken focused on three primary care trusts and their partner organisations. | NIHR publication | Grey literature (case studies) |
| Uncovering the processes of knowledge transformation: the example of local evidence-informed policy-making in United Kingdom healthcare | 2020 | Gabbay, J.; le May, A.; Pope, C.; et al | To understand how and why healthcare policy-makers transform research-based evidence into the knowledge they ultimately use. | Interviews, meeting observations and document reviews in four healthcare-commissioning organisations working with external agencies | Journal article, peer-reviewed research | Qualitative research (multimethod) |
| Understanding primary care co-commissioning: Uptake, development, and impacts. Final report | 2018 | McDermott, I.; Warwick-Giles, L.; Gore, O.; et al | To understand the scope of co-commissioning activity and the process of change, the practice of co-commissioning, to understand how does co-commissioning affect the external relationships of the CCG, what (if any) new or altered services have been established as a result of co-commissioning, and what factors facilitated or inhibited the development of new services or the improvement in existing services | Interviews with senior policy makers, review of documents, telephone surveys and case studies | Report (PRU Comm) by PRU Comm, a collaboration between London School of Hygiene and Tropical Medicine, the University of Manchester, the Centre for Health Services Studies at the University of Kent and the Personal Social Services Research Unit. | Grey literature (mixed methods) |
| Understanding the new commissioning system in England: contexts, mechanisms and outcomes | 2018 | Checkland, K.; Hammond, J.; Sutton, M.; et al | To assess the impact of the reforms on the operation and outcomes of the commissioning system. | Analysis of publicly available policy documents, case studies of two local areas of England including interviews, quantitative analysis of relevant outcomes | This report is based on independent research commissioned and funded by the NIHR Policy Research Programme ‘Understanding the new commissioning system in England: contexts, mechanisms and outcomes’ PR-R6-1113-25001 | Grey literature (mixed methods) |
| Uptake of NHS health check: issues in monitoring | 2018 | Riley, V.; Gidlow, C.; Ellis, N. | To explore the perceptions of those involved in commissioning of NHSHC to better understand the implications for local and national monitoring and evaluation of programme uptake. | Interviews with NHSHC commissioners and leads | Journal article, peer-reviewed research | Qualitative research (interviews) |
| Using clinical practice variations as a method for commissioners and clinicians to identify and prioritise opportunities for disinvestment in health care: a cross-sectional study, systematic reviews and qualitative study | 2015 | Hollingworth, W.; Rooshenas, L.; Busby, J.; et al | To explore if geographical variation in procedure rates is a marker of clinical uncertainty and might be used by local commissioners to identify procedures that are potential candidates for disinvestment. | Analysis of Hospital Episode Statistics to measure geographical variation in procedure rates, rapid systematic reviews of two high-use procedures selected by the PCTs, non-participant overt observations of commissioning meetings and semistructured interviews with stakeholders about disinvestment in general and with clinicians and patients about one disinvestment case study | NIHR report | Grey literature (mixed methods) |
| Using RightCare to engage general practice nurses in Liverpool | 2017 | Poll, S.; Lloyd, K | To describe an approach used by Liverpool Clinical Commissioning Group. The CCG is using the RightCare approach to engage nurses working in its 92 GP practices, prompting them to discuss what can be done to improve care and reduce variation | Description of a case study | Journal article, peer-reviewed research | Descriptive piece |
| Using routine data to conduct small area health needs assessment through observing trends in demographics, recording of common mental health problems (CMHPs) and sickness certificates: longitudinal analysis of a northern and London locality | 2010 | Chan, T.; Cohen, A.; de Lusignan, S. | To examine the trends in common mental health problems (CMHPs), prescription of antidepressant, anxiolytics and hypnotics and medical certificates over four years to highlight the strengths and pitfalls in trends analysis using primary care data. | Relevant clinical information for the first six months of each of the calendar years 2004, 2005, 2006 and 2007 were extracted from participating practices in a London locality and in the North | Journal article, peer-reviewed research | Longitudinal study |
| Using routinely gathered data to empower locally led health improvements | 2013 | Dhillon, A.; Godfrey, AR. | The authors suggest that routinely gathered data should also be used to help clusters of practices to learn from locally led innovation and to motivate long-term partnerships for interorganisational health improvement. The authors describe some of the existing data sources that could be used to do this as well as some of the dangers of using data in this way | The authors have roles as GPs, Health Network members and Clinical Commissioning Group leaders. In this article they describe how, in their opinions, routinely gathered data to empower locally led health improvements | Journal article, peer-reviewed research | Descriptive piece |
| Using routinely gathered data to evaluate locally led service improvements | 2011 | Stoddart, G.; Gale, R.; Peat, C.; et al | To describe a test done by NHS Ealing, whereby they tested the feasibility of a) combining data from more than one data-domain at the same time to quantify patient movement across the primary care/acute hospital boundary, and b) establishing online analyses so they can be constantly updated with near real-time data to compare different subsets of patients. In addition, to describe how the reports gave the teams leading the projects confidence in the projects and helped to influence local policy. | Description of an intervention run in the NHS in Ealing | Journal article, peer-reviewed research | Descriptive piece |
| Views of NHS commissioners on commissioning support provision. Evidence from a qualitative study examining the early development of clinical commissioning groups in England | 2014 | Petsoulas, C.; Allen, P.; Checkland, K.; et al | To explore the attitudes of CCGs towards outsourcing commissioning support functions during the initial stage of the CCG reform | Case study research design in eight CCGs, conducting in-depth interviews, observation of meetings and analysis of policy documents. | Journal article, peer-reviewed research | Qualitative research (case studies) |
| We can't get anything done because...': making sense of 'barriers' to Practice-based Commissioning | 2009 | Checkland, K.; Coleman, A.; Harrison, S.; et al | To investigate the issues raised by participants as 'barriers' to the development of Practice-based Commissioning (PBC) in 'early adopter' sites in England. | Case studies of five PBC consortia in three Primary Care Trusts (PCTs). Data collection included interviews, observation of meetings, and analysis of documents tabled at meetings and circulated at other times. | Journal article, peer-reviewed research | Qualitative research (case studies) |
| What antimicrobial stewardship strategies do NHS commissioning organizations implement in primary care in England? | 2020 | Allison, R.; Lecky, DM.; Beech, E.; et al | To explore the adoption and use of education/training and supporting AMS resources within NHS primary care in England. | Analysis of questionnaires sent to the medicines management teams of all 209 Clinical Commissioning Groups (CCGs) in England | Journal article, peer-reviewed research | Survey analysis |
| What do external consultants from private and not-for-profit companies offer healthcare commissioners? A qualitative study of knowledge exchange | 2015 | Wye, L.; Brangan, E.; Cameron, A.; et al | To learn about the support offered to healthcare commissioners, how external consultants and their clients work together and the perceived impact on the quality of commissioning. | Mixed methods case study of eight cases across NHS commissioning organisations and private and not-for-profit providers, including interview, observation of training events and meetings, as well as document analysis | Journal article, peer-reviewed research | Qualitative research (case studies) |
| What happens when GPs engage in commissioning? Two decades of experience in the English NHS | 2016 | Miller, R.; Peckham, S.; Coleman, A.; et al | To review the evidence on commissioning schemes involving clinicians in the United Kingdom National Health Service, between 1991 and 2010 | Secondary research - review of published evidence | Journal article, peer-reviewed research | Literature review |
| Where next for commissioning in the English NHS? | 2010 | Smith, J.; Curry, N.; Mays, N.; et al | To example the development of NHS commissioning and set out practical suggestions for how it might be strengthened to secure more effective and efficient care. | Secondary research - literature review | Grey literature (published jointly by The Nuffield Trust and The King’s Fund) | Grey literature (literature review) |
