# Supplementary material for: The usage of data in NHS primary care commissioning: a realist review
Source: BMC Med. 2023 Jul 3;21:236. doi: 10.1186/s12916-023-02949-w (PMC10318817; doi:10.1186/s12916-023-02949-w)
Supplement: Supplementary file 5 — Additional file 5. Final programme theory. [file 12916_2023_2949_MOESM5_ESM.docx]

The 30 CMOs representing the final, revised programme theory are presented on pages 2 to 6. They are grouped by conceptual category and linked back to the initial programme theory (additional file 2). Examples of supporting quotes from the included studies and, where applicable, the substantive theories used to inform CMO development are shown on from page 7 onwards.


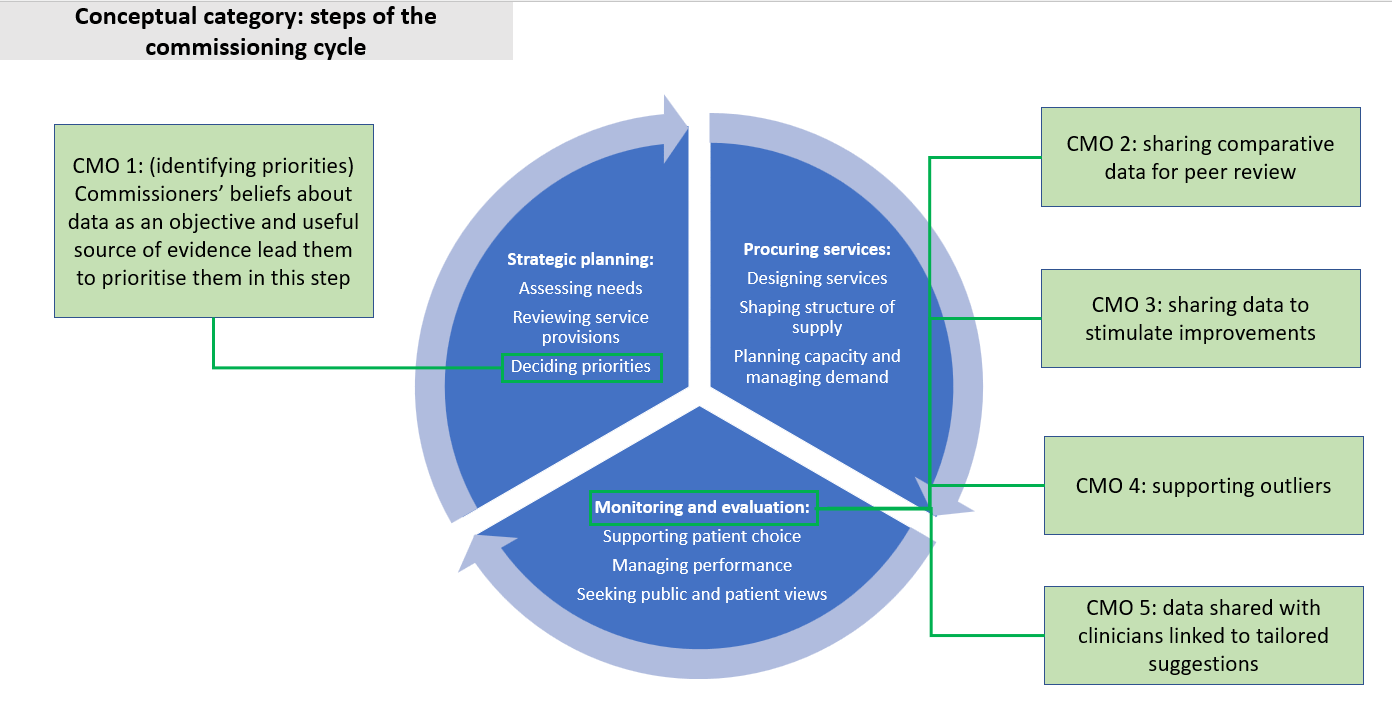


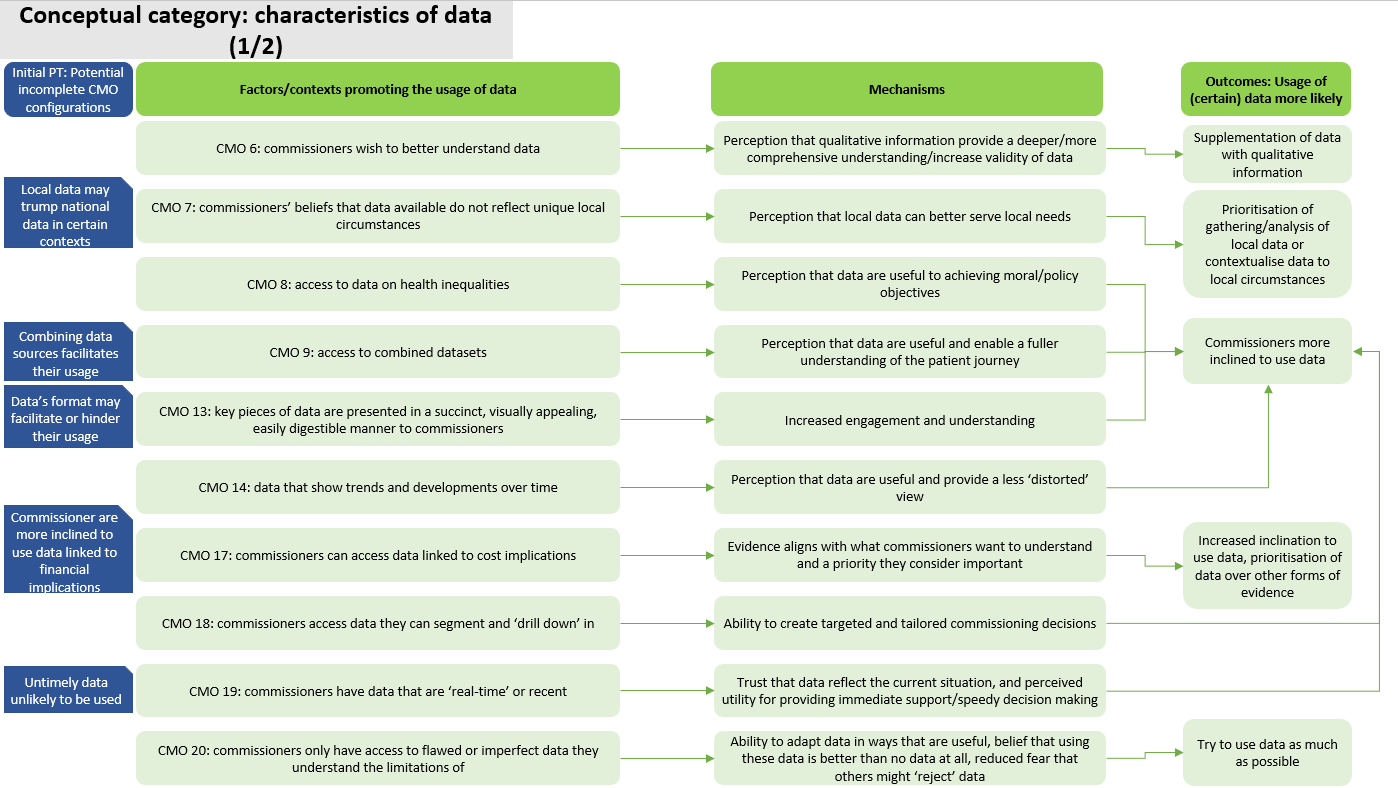


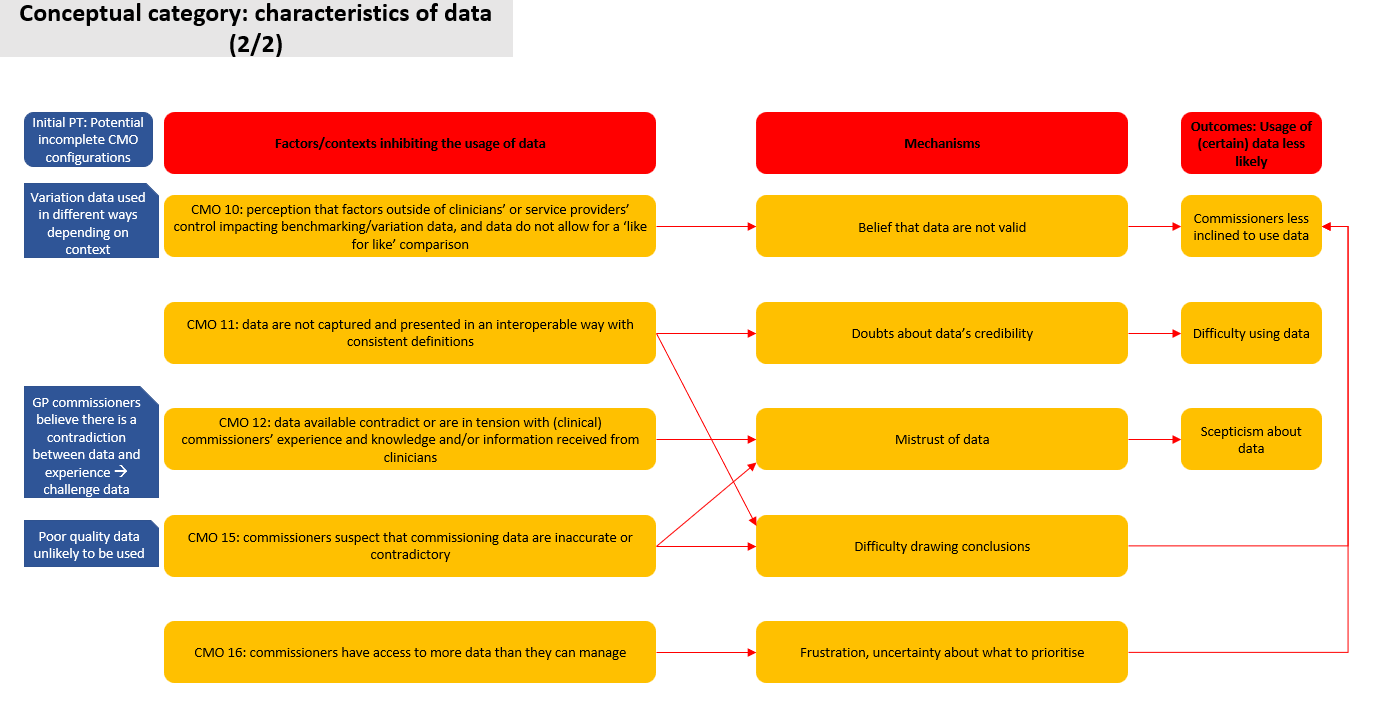


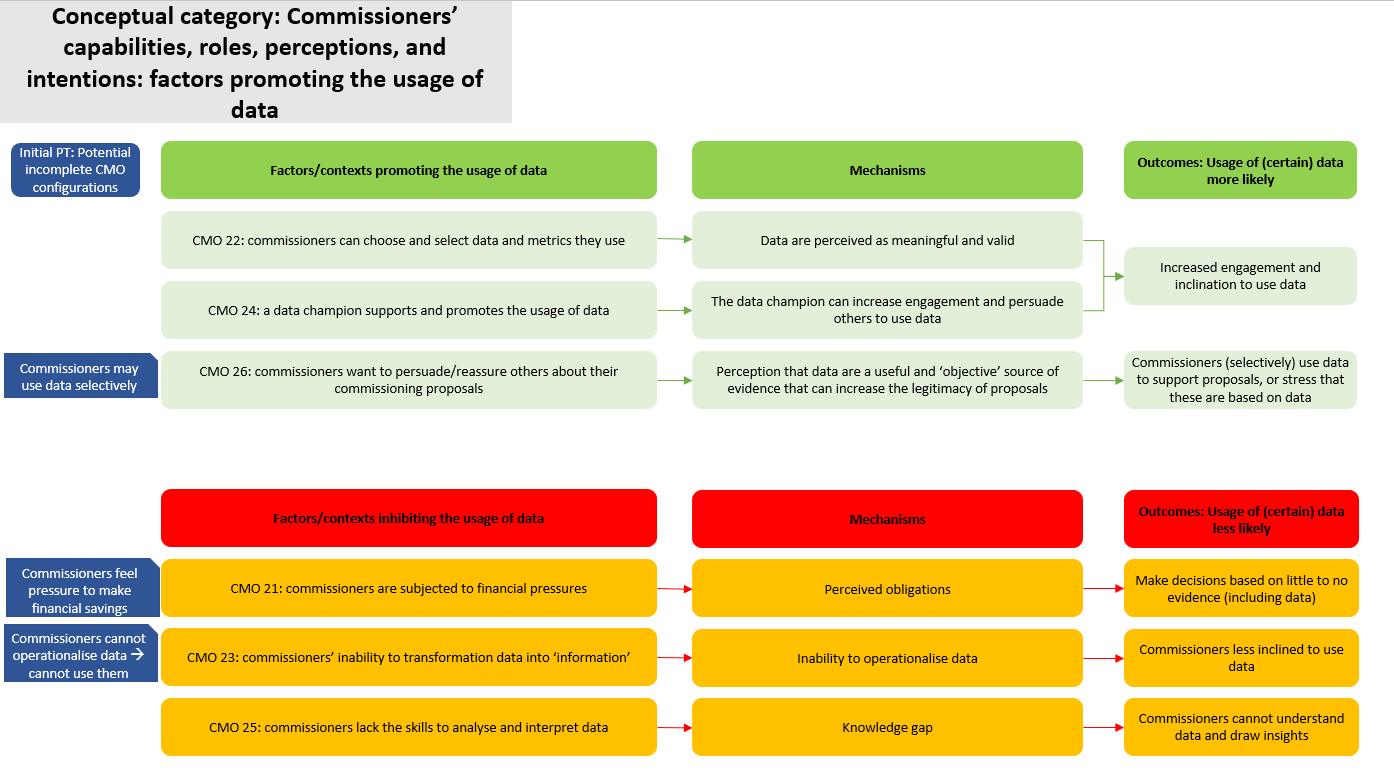


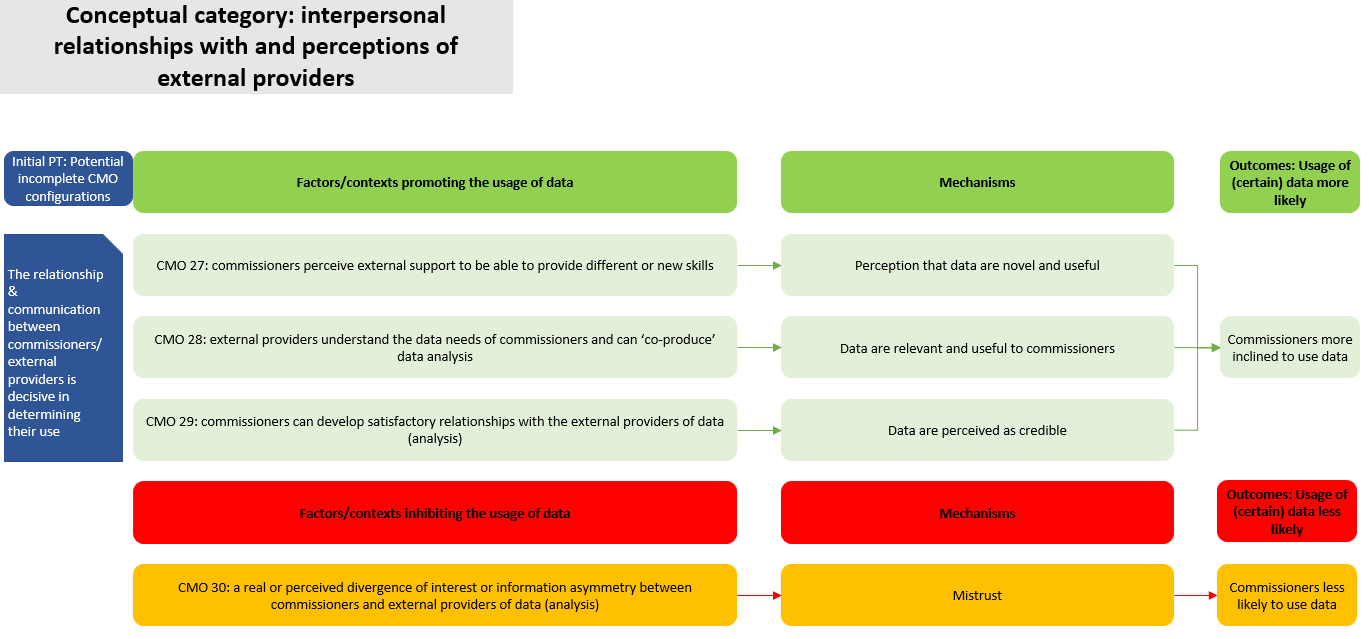


| **Number** | **CMO** | **Substantive theories facilitating CMO development** | **Sample supporting quotes (italics denote direct speech)** |
| --- | --- | --- | --- |
| **Category: steps of the commissioning cycle** | | | |
| CMO 1 | When commissioners wish to use an ‘objective’ source of evidence to identify commissioning priorities (C) commissioners will be inclined to use data (O) because commissioners view data as a neutral and impartial source of evidence (M) | Counting or quantification (Deborah Stone). CMO 1 was informed by the concepts related to ‘counting’ or quantification or policy issues as outlined by Deborah Stone in her work ‘The Policy Paradox.’ | Furthermore, in a presentation prepared for the SHA, which would evaluate the quality of the SP, the PCT demonstrated confidence in that:  *‘We are taking a data driven approach in the strategy refresh. In the first stage we took an objective look at the data. Based upon the data we then did further analysis on the big issues that emerged, to determine a prioritised issue short list. The prioritisation tool identified a number of potential strategic issues... we then prioritised initiatives and selected a limited number of initiatives for further development... The Strategic Issues group into three Themes.... (The third issue is to) ‘Improve long term condition management’, (with priority strategic issues: respiratory disease, CHD, Stroke, Heart Failure, Diabetes, CVD).*(Swan et al., 2012)  A striking characteristic of the background papers and oral summary is the way in which policy problems are constructed in terms of quantitative information, with an emphasis on numerical patterns, quantities, and levels. The starting point for exploring a particular policy problem is typically ‘looking at the data’. So in the case of an agenda item about hospital discharge of elderly people (an agenda item titled ‘hospital bed cost savings proposal: enhanced planning of the whole systems approach’) the starting point of the background paper is an analysis of health resource group ‘trim points’ (the point at which additional costs per day are incurred if a patient exceeds the average anticipated stay for a particular condition or procedure). The following extract, reported in full to convey this emphasis on numerical information, is taken from the author’s summary of the background paper, introducing the item to the Priorities Forum:  *‘So what we did was, was to go back and have a look at the data. And basically, what we did, was to run off – erm, the information, is now stored on our Information Systems…’*(Russell and Greenhalgh, 2009) |
| CMO 2 | When commissioners believe that clinicians will act to improve when comparative data are shared and scrutinised by peers (C) they will give them data so they can review and compare their performance relative to their peers (O) because they believe improvements will occur when; there is peer pressure and orientation to a reference group (M); competition (M) and; clinicians are more receptive to feedback from their peers (M) | Reference group theory | In two PCTs, for example, maps of variation showing less than 30% of patients with diabetes had received nine key care processes, as opposed to over 70% in the ‘best’ PCT, helped to convince general practitioners that not only performance was unacceptably poor, in relative and absolute terms, but also that improvements were possible. PCT staff perceived the NHS Atlas as a *“catalyst which motivated clinicians to take action sooner than they might have done otherwise”* (Director of Commissioning, PCT22).(Schang et al., 2014)  Another CCG chairperson recounted two examples of behavioural change due to peer review. Once was when data suggested that his own behaviour with X-ray referrals meant that his practice was the second highest user of X-ray services in the CCG, and the other instance was with a GP colleague.  *We’ve got a GP who has been known about by the PCT for years, who spends exactly twice as much on drugs as anyone else in our group. [PCT chief executive] would go round and plead with him. He’d be very polite, give him tea, coffee, biscuits, anything he liked, but what really mattered [was] controlling his prescribing expenditure. Since he’s had peer review, and charts up on the wall showing where he is with his peers, he’s accepted mentorship from one of our GP leads, you know, and he’s starting to make some progress. In other words, he’d flick a V sign at the, you know, the managers, but he doesn’t do that to his peers.*  Martin, CCG chairperson(Wye et al., 2015a)  All except three of the pilots reported having secured professional prescribing advice for the group. The most frequently reported role of the prescribing adviser was that of collating, analysing, and reporting on pilot prescribing data. This information was typically fed into a programme of targeted visits by the adviser or pilot lead GP to practices deemed to be ‘outliers’ according to a range of quality or financial markers for prescribing.  The open sharing of these prescribing data within pilots, on a named individual practitioner basis, was cited as a key issue by 23 pilots. Whether or not to name doctors when sharing prescribing data had been an issue for many groups and nine pilot groups specifically reported that they had moved to a position where they felt that there was sufficient mutual trust within the group for this to be possible.(Smith et al., 2000) |
| CMO 3 | If commissioners believe that clinicians or service providers are not aware their performance is below average (C) and they do not want to be perceived as ‘judgemental’ or ‘performance managing’ they may share data with them in the hope of stimulating improvements (O) because of a perception that sharing data can empower clinicians or service providers to come up with solutions (M) |  | The NHS Comparators application, which was launched last year, provides comparative data from SUS to enable commissioners and providers to investigate aspects of local activity, costs and outcomes.  For practice-based commissioners, it is enabling them to review the way they refer patients into secondary care, highlighting areas where referrals are out of line with local, regional and national patterns.  It has had a significant impact on the front line. For example, it prompted an Essex GP to review how his practice was handling ear, nose and throat patients because referrals for that specialty were much higher than average. A GP practice in Staffordshire launched a special, evening clinic for asthmatics after NHS Comparators highlighted an excessively large number of its asthmatic patients going to hospital for urgent care. NHS Comparators now contain new indicators to help frontline staff meet the 18-week target by identifying potential problem areas.(Dewhurst, 2008)  On the positive side, and in line with ﬁndings of the Audit Commission report (2007), the NPCRDC report showed that the sharing of performance data had begun to change GPs’ behaviour, making them more aware of their own performance and that of their peers. In addition, more GPs were aware of the cost implications of their referral behaviour, and were willing to change as a result (Checkland et al 2008).(Curry et al., 2008)  *‘Can be a real wake-up call when your CCG realises they are very high prescribers compared to other areas.’* (CSU-23— Questionnaire)(Allison et al., 2020) |
| CMO 4 | Where data indicate potential for improvement, and commissioners suspect that clinicians need help to achieve improvements (C) and commissioners want to be seen as supportive rather than judging (C) commissioners may offer support to outliers and underperformers (O) because they want to maintain good relationships by being perceived as supportive (M) |  | Most areas reported having an audit process in place that identified outliers for targeted support. Outcomes from these audits included a reduction in prescribing levels, improved partnership understanding of the issues and in some areas, increases in dedicated POM/OTC medicines treatment provision.(NHS National Treatment Agency for Substance Misuse, 2011)  At the governing body level, the majority of the CCGs in this research used comparative practice data as a way of identifying practices that were outliers in terms of their performance, for example for referral or prescribing rates. The intention is then that CCGs could instigate a process of providing peer support (for example through joining up highly performing practices with those that are outliers) or practice visits by CCG leaders to identify how improvements could be made. In one CCG, if this process does not work, it has a policy by which the under-performing practice would be asked to explain itself to the members’ council (this had not been used to date). None of the CCGs had set performance objectives for practices (unless they were used to monitor specific financial incentives). Some CCG leaders were reluctant to be seen to be setting performance targets out of concern that it would be taking GPs back to the days of PCTs and disengage the membership: *“I’m loath to use those kinds of words [performance management] because they [the membership] will roll their eyes and say, ‘here we go, it’s all over again’. So it’s a really fine line we have to tread.”* (Chair)(Holder et al., 2015) |
| CMO 5 | When commissioners share data with clinicians that is easy to understand and linked to tailored suggestions for action (C) it increases clinician engagement (O) because clinicians understand how to make improvements (M) |  | *‘Unfortunately, these letters get sent out without CCGs being aware of this. Quite often it is unclear why practices have been sent letters as the data we have does not suggest they should have been targeted. Our highest prescribing practices often do not receive these letters. As we have not been involved with this process it is hard for us to explain why the practice has received the letter and what they need to do. I therefore think this is unsuccessful as it disengages the practice. If this process was done with the CCG teams I think this would improve the process - we could work together to get the letters to our least engaged/highest prescribing practices and then follow up with an offer of support tailored to meeting the requirements of the letter.’* (CCG-96 – Questionnaire)(Allison et al., 2020)  Our intervention incorporated a range of evidence- and expert-informed suggestions to improve the effectiveness of feedback, such as providing repeated feedback with comparators to reinforce desired behaviour, recommending specific actions, and ensuring credibility of information(Alderson et al., 2021)  A learning health system is a system that is aligned for continuous improvement through the assembly of data from various sources, the analysis of the data and regular feedback of findings to instigate a change in practice. The UK government stated as part of their 5- year national plan to reduce AMR a need to ‘Use electronic prescribing data to give healthcare providers feedback on guidance compliance and prescribing rates’. The current project aimed to build an interoperable infrastructure that can provide feedback to general practices on their antibiotic prescribing, which is tailored to their characteristics and is independent of the software system as used by the practices (most general practices in the UK use one of three). This infrastructure will analyse antibiotic prescribing and patient characteristics at fortnightly intervals and compare the results of the participating practices to comparable data from large national datasets (the Clinical Practice Research Datalink and the Secure Anonymised Information Linkage (SAIL) Databank) in order to provide peer comparisons and deliver tailored results back to each practice.(Palin et al., 2020) |
| **Category: characteristics of data** | | | |
| CMO 6 | In contexts where commissioners wish to better understand data (C) they may supplement data with qualitative information (O) because of the perceived increase in the validity of data (M) and due to a perception that this will give them a fuller and more meaningful understanding of the data (M) | Mixed methods research. CMO 7 was informed by the framework of mixed methods research and the mechanisms it purports to activate, e.g. increased validity, deeper understanding of data, etc. | *“...We need more about the impact of oral health on quality of life...where they [patients] place the priorities for management of their conditions. So it would be more of the qualitative side I would like to see”.* [P3, consultant, male].(Holmes et al., 2009)  Qualitative information proved to be a valuable addition to the data. From the perceptions of practitioners in particular it appeared they had a common understanding of the problems among school children and they fulfilled well the function of filling gaps in the data and validating statistical information.(Billings, 1996) |
| CMO 7 | In a context where commissioners believe that data do not reflect unique local circumstances or cannot be applied locally (C) they may prioritise gathering or analysis of local data (O) or attempt to contextualise data to local circumstances (O) due to the perception that this can better serve local needs (M) |  | *‘You can’t use national statistics to drive what we’re doing locally because our patients have different needs and we need to build data up from the ground asking ‘Well, what do we need for our patients? How should that look? How will it serve them best from a clinical perspective?’*(Currie et al., 2018)  Contextualisation involved taking information from elsewhere and applying a local lens or filter. Commissioners had an essential role in contextualising the knowledge to local circumstances, but sometimes external providers had to undertake contextualisation as well so that the knowledge was fit for commissioners. When appropriating information from elsewhere, one commissioning manager commented that someone *‘always says our system is not like that’* (Clara, NHS commissioning manager), and so the knowledge needed to be contextualised to overcome this hurdle. One commissioning manager said that contextualisation was the ‘crux’ of commissioning.(Wye et al., 2015a)  Local data often trumped national or research-based information and local evaluations were seen as helpful in directly answering commissioners’ questions.(Wye et al., 2015b) |
| CMO 8 | When commissioners have access to data on health inequalities (C) they may perceive them as useful to achieving a policy and/or moral objective (M) making commissioners more likely to use the data (O) |  | *[The contracting department] need to be knowledgeable about aspects of ethnicity and should get into a mode of thinking about all populations, not just the white middle classes, which has been the tradition in the NHS and I think that's not happening.*(Evans, 1999)  In terms of understanding population health needs and identifying gaps in provision, most respondents emphasised the central importance of good ethnic monitoring data, particularly in primary care, as this was felt to offer a population-level ethnic proﬁle.(Salway et al., 2013) |
| CMO 9 | If commissioners have access to combined datasets (C) they will be more likely to use them (O) because the data are useful (M) and because commissioners can gain a fuller understanding of the patient journey (M) |  | It was argued that in an ideal world, a fully costed and commissioned service would have one central point of contact that would manage the patient’s data and journey, seamlessly, around the spoke services.(Storey et al., 2018)  This is linked with comparative data across the PCT and the Strategic Health Authority, and can also link with national data. it not only profiles the practice but, by using your practice demographics for inequalities and other things, it can also help you make comparisons with practices that you can see are of a similar “cluster” profile.(Shepherd, 2009)  As more data are available and able to be linked at person-level, commissioners want to analyse demand (public health and epidemiology data, together with information about service utilisation) in far more detail.(Smith et al., 2010)  It was argued that in an ideal world, a fully costed and commissioned service would have one central point of contact that would manage the patient’s data and journey, seamlessly, around the spoke services.(Storey et al., 2018) |
| CMO 10 | In a context where commissioners feel that factors outside of clinicians’ or service providers’ control are impacting benchmarking or variation data (C) or the data do not allow for a ‘like for like’ comparison (C) they will be less included to use the data (O) because they think they are not valid (M) |  | *‘... we might do some decision making around prescribing levels practice by practice then you might say the expected levels of prescribing of statins if you’re living in an area where you’ve got, a higher incidence of, of CHD (Coronary Heart disease) you might look at... that practice has a higher than normal population of that then you might look at individually what there prescribing rates are and what they are doing and say well the two don’t match... It’s too low for the expected population... we do that... don’t we...’*(Chinamasa, 2007)  Performance comparisons between CCGs were felt to be legitimate, as long as difference between populations was recognised in the process:  *So, if you’re going to be benchmarked, let’s get the right peer groups, and be really quite savvy about that. So, if you are in an urban, deprived city, with a high Asian population, let’s make sure you benchmark against a very similar city, whether that’s [city w] or [city x], or somewhere, but don’t start benchmarking against [city y], ‘cause it’s very, very different. Or even [city z] is very different isn’t it, and so it’s about being really clear that where you’re looking at, at health that is largely influenced, or things that can influence, are around, sort of other things, like your ethnic mix, and your depravation, that you bench that, mark that properly.* 21891, CCG manager, Area 2(Checkland et al., 2018)  You can also look at the dataset of how you are performing on quality and outcomes framework data. the profile compares how you treat your diabetics not only in Calderdale and across the SHA and nationally, but also with your cluster practices. You are comparing like with like so there is no excusing yourself.(Shepherd, 2009) |
| CMO 11 | When data are not captured and presented in an interoperable way with consistent definitions (C) commissioners will have difficulty using them (O) because they have difficulty drawing conclusions (M) and doubt the data’s credibility (M) | Theory of interoperability | The new CSU manager described difficulty in sourcing and evaluating the data:  *It depends how it is coded though. So every intervention in a hospital is recorded and coded . . . it is just a matter of deciphering what it is that is relevant and what is not. So someone might come in with a stroke and it might not necessarily say they have got diabetes but it could have been the diabetes that caused the stroke . . .*  CSU project manager(Swan et al., 2017)  When asked about national NHSHC data, some participants believed there is ‘variation with how people are reporting’ (p. 7), which reduced the perceived credibility of national data: ‘I’m not 100% sure that you’re comparing like with like’ (p. 7). For two commissioners, inconsistencies in reporting opportunistic invitations and relying on self-reported data led them to believe the national data ‘may be skewed either negatively or positively by inconsistent coding’ (p. 8). Others thought it was ‘very limited what they [Public Health England] expect back from us’ (p. 11) and it ‘doesn’t tell you anything about the quality’ (p. 12). These participants believed that more data should be reported, such as  ‘patient demographics’ (p. 6), to better understand the national NHSHC population.(Riley et al., 2018) |
| CMO 12 | In a context where (clinical) commissioners find that the data available contradict or are in tension with their experience and knowledge (C) commissioners may become skeptical of the data (O) because they are mistrustful (M) |  | *. . . the CCG have done a piece [of research saying] 70% of the activity can be stripped out . . . we’re challenging that . . . I’ve spoken to a GP colleague [who had] one patient that she felt could be managed in their practice . . .*(Swan et al., 2017)  We regularly observed clinical input to commissioning decisions in observations of CCG meetings. For example, in one CCG the group were looking at an unscheduled care dashboard to attempt to identify patient groups that could be diverted from hospital. Two GPs had prolonged discussion about cellulitis, applying their clinical knowledge to interpret information from the dashboard.(Wye et al., 2015a) |
| CMO 13 | Presenting key pieces of data in a succinct and easily digestible manner to commissioners (C) can increase the likelihood they will be used (O) since this increases their engagement with and understanding of data (M) |  | The first noticeable impact of their participation on the contract monitoring process was the presentation of the data at the monthly meetings. Rather than ‘raw’ activity figures being tabled on the day of the meeting, a comprehensive activity report was circulated to all members at least seven days before the meeting. This reporting was considered to be a definite improvement on the previous system. The circulation of the report before the meetings and the manner in which the data was presented offered people enough time to read, digest and prepare any comments before the meeting.(Cowie, 2002)  Documentation was often sent electronically. Documentation included performance, activity, financial and referral data from a range of health-care providers, directives and guidelines from the Department of Health and regional bodies, meeting papers, business cases, reports, patient satisfaction surveys, guidelines and pathways. Often key points were summarised on a side of A4 or an executive summary, possibly because the volume of reading was unmanageable. For example, governing board members had usually at least a dozen documents of several pages each to read before monthly meetings. Presumably to make this task easier, many documents had standardised cover sheet with information such as title, purpose of the paper and action required. In some cases, executive summaries of one page or shorter directly followed these cover sheets.(Wye et al., 2015a)  One E&D lead with very little statistical experience created a simple spreadsheet in Excel that took data from a publicly available GP satisfaction survey and compiled individual-level GP practice scores to provide a picture of local satisfaction by ethnicity. This information was presented in a succinct and meaningful way and was fed into the Equality Delivery System. This led the strategic health authority to fund a further data analysis exercise to break down information from national GP and hospital patient surveys by protected group.(Salway et al., 2013) |
| CMO 14 | Where commissioners have access to data that show trends and developments over time (C) they will be more inclined to use them (O) because they consider this useful (M) and think this will provide a less ‘distorted’ view (M) |  | Data drawn from constantly changing situations can be misleading because of the changing denominator. Because of patients moving on and oﬀ the practice list, a practice with a particularly mobile population may have immunised more than 100% of their practice population but still have 10% of children without immunisations, whereas another practice with a more stable population may have immunised 99% and only have 1% without immunisation. Data catches a snapshot of more complex stories-in-evolution.(Dhillon and Godfrey, 2013)  In addition to the difficulty in getting hold of some of the primary care contracts from NHSE, access to historic data is another issue brought up by our case study CCGs:  *Well for example the CQRS system which is a system that records the data for QOF and for the DESs that will subsequently trigger the payments for that and that’s something that practices are really interested in of course. As a CCG we have been unable to access that, with effect from the 1st of April this year 2016 we can now access CQRS, however we can't access historic data so there’s no ability for us to have a look at trends, have a look to see how things have changed and we have to ask NHS England for all of that information. For the QOF signoff we had to ask for all of the background information to be able to authorise all of the payments. There’s a lot of information still held by NHS England that we don't have direct access to and that’s not necessarily anybody’s fault it’s the way the greater system works and it’s data, it’s access to data that is very frustrating and having to ask people who are busy doing their own work to provide data for us to do our work is...can be uncomfortable when you know how busy they are. So there needs to be and we’ve tried to push for a change to the CQRS system so that we can access data from the 1st of April 2013 which would be really useful to us and unlock a lot of the other systems like that. It’s the systems it’s not the people, I know the people are working as well as they possibly can, the systems don't allow it.* [Manager ID37](McDermott et al., 2018) |
| CMO 15 | In a context where commissioners suspect that commissioning data are inaccurate or contradictory (C) they will not use them in commissioning decisions (O) because they do not trust them (M) and have difficulty drawing conclusions (M) |  | A more common story, though, was of a lack of accurate information about potential demand to inform service redesign:  *‘But this is the biggest handicap within the NHS, is accuracy and completeness of data. And be able to look at a number and go, ‘That’s the number.’ And not look at six different numbers.’*(Porter et al., 2013)  Inappropriate and poor quality data, a lack of robust information systems and capacity to generate data and interpret knowledge were identiﬁed as considerable hurdles(McCafferty et al., 2012) |
| CMO 16 | In a context where commissioners have access to more data than they can manage (C) they may feel frustrated (M) and uncertain about what to prioritise (M) meaning they are less likely to use the data (O) |  | One respondent encapsulated this view about data ‘‘overload’’:  *The ready availability of information and community views has been a problem. There is much information in the system that we cannot quickly use* (Joint Local Authority/PCT submission, 14).(Ellins and Glasby, 2011)  Commissioners and clinicians frequently described problems with the production of knowledge and information overload. The challenge of multiple sources of information compiled by national bodies is signiﬁcant and can mean that the practical utility of evidence is lost.(Shepperd et al., 2013)  *I think that what would be critical is having the information available. What we don’t have as a committee is the resources to be able to go and ﬁnd the information and put it together, assess it to make sure that it’s robust and then deliver it to the committee. We don’t have the team to be able to do that sort of work.*(Williams and Bryan, 2007) |
| CMO 17 | In a context where commissioners have access to data linked to cost implications (C) they will be more inclined to use them (O) and may prioritise the data over other forms of evidence (O) because the evidence aligns with what they want to understand (M) and because it aligns with a priority they consider important (M) |  | The PEC [professional executive committee] members need to know what outcomes they want and the ﬁnancial effects or health impacts these will have when making suggestions.(Offredy, 2005)  *“What I want to know is: where do we have the largest potential for efﬁciency savings, that don’t harm patients . . . the Atlas alone can’t tell me that”* (Financial Director, PCT12)(Schang et al., 2014)  *“And until you actually start understanding, not spend, but cost, and actually understanding the pathway, and so some of that … and I think that’s where the QI methodology learning has come in, you know, there’s something about you really need to understand where the resource has gone and what did you get for it”* (CCG2, Commissioner 1: CCG, GP)(Moran et al., 2018) |
| CMO 18 | When commissioners have access to data they can segment and ‘drill down’ in (C) they are more inclined to use the data (O) because they are able to create targeted and tailored commissioning decisions (M) |  | *[G]eneral practice information is the best contemporaneous source of information that we have on the*  *population and the health of the population [… I] t’s much better if that can also be segmented, not just by health condition or whatever, but by population group.* (Interviewee 10, Public Health).(Wyatt et al., 2018)  Having these data and being able to disaggregate by specific variables renders it ‘the best contemporary source of information we have on the population and the health of the population’.(Wyatt et al., 2018)  *Some of the data that we get is very difficult to scrutinise . . . It’s a very aggregated data set so it’s very difficult to try and actually drill down into that data to see whether you really think there’s anything wrong . . . we can’t drill down.*(Currie et al., 2018) |
| CMO 19 | When commissioners have data that are ‘real-time’ or recent (C) they will be more inclined to use them in commissioning decisions (O) because they find them useful for providing immediate support or enabling speedy decisions (M) and they have trust that the data reflect the current situation (M) |  | The development of comprehensive real-time linked data systems, including data from primary care as well as other sources, will enhance surveillance of a wide variety of health threats and improve our ability to react quickly and effectively to protect the health of the population.(Department of Health, 2006)  Commissioners in this research frequently called for better and more timely data about local needs, services, costs of provision, and patients’ and carers’ experience. There was a strong perception of such data being in existence and used for needs assessment and service specification, yet problematic to access and use in real-time for review and performance management with providers.(Smith et al., 2013)  *‘I’ve experienced some frustration with actually getting data, the timeliness of information being produced. I think because it’s such a huge organisation looking after so many CCGs sometimes the prioritisation as a customer you start thinking well I can’t move a piece of work. I’ve waited three and half months recently for some ultrasound data and we had to escalate it, letters coming from the network to get something happening because there just didn’t seem to be any impetus and we were just being, well what I felt was fobbed off, we need to do this, we’ve got to do that, which doesn’t help’* [GP ID 132].(Checkland et al., 2014) |
| CMO 20 | If commissioners have access to flawed or imperfect data they understand the limitations of (C) and this is the only type of data they have access to (C) they will still try to use the data (O) because they can adapt them in ways that are useful while taking into account the data’s limitations (M) and they believe this is better than using no data at all (M) |  | *I know people often feel that absence of data or poor data quality are obstacles, that they can’t address the particular question they’re interested in. My perspective has always been slightly different. I just think that you’ve got a question, you’ve got to make that decision. You deploy whatever data you’ve possibly got at that time in whatever form you’ve got it to answer that question as best you can. There’s no point just shrugging and saying you can’t do anything . . . we have to go with what we’ve got really and part of our job is knowing how to make best use of the data sets that we already have, thinking of different creative ways of using the data to answer the questions they’ve got and knowing which fields or records we should just ignore because we know the data quality’s not sufficiently robust.* CCG M, interview 11(Currie et al., 2018)  For instance, some respondents pointed out that ethnic monitoring data is often more complete than might be imagined and that there are strategies that can be used to work around incomplete data and produce useful information.(Salway et al., 2013) |
| **Category: commissioners’ capabilities, roles, perceptions, and intentions** | | | |
| CMO 21 | In a context where commissioners are subjected to financial pressures (C) they may choose to make commissioning decisions based on little or no evidence (including data) (O) because they feel obliged to prioritise financial issues (M) |  | Moreover, sometimes initiatives with negative or no evidence were funded because commissioners still needed to deliver a viable financial plan. The imperative to come up with an initiative that looked like it could save money would push it through, regardless of research that suggested the contrary (or no research at all).  *I’ve had conversations [with colleagues] about, “Well, you know, we shouldn’t be putting that down to say it will make savings because there’s no evidence that it will,” versus me saying, “But actually we’ve still got a statutory responsibility to deliver a balanced plan, and if I take those savings out they need to come from somewhere else.”* (Carla, NHS commissioning manager, Norchester)(Wye et al., 2015b)  *“You’ve got to take your purchasing decisions on the best information and research you can get. But in the end they’re dictated by corporate priorities, ﬁnancial priorities and hopefully informed by that kind of independent viewpoint on what you knew”*(Elliot and Popay, 2000) |
| CMO 22 | In a context where commissioners can choose and select data and metrics they use (C) they will become more engaged and inclined to use the data (O) because they are meaningful and valid to them (M) |  | English GPs valued monitoring data only when they had selected information that would be meaningful and useful to them.(Rod et al., 2015)  The majority of participants thought, ‘an awful lot of emphasis [nationally] is from the uptake percentage’ (p. 15), which some considered ‘a meaningless statistic’ (p. 5). There were calls for more focus on ‘how many are eligible and, of those, how many have had a health check’ (p. 5) in the last five years (known as coverage) instead of uptake (percentage of those invited and received a NHSHC). Participants also identified that success of NHSHC varies when uptake is used as a performance indicator: ‘if the local authority uses an opportunistic only model then their uptake is going to be very high vs someone who uses the call and recall system’ (p. 15). Overall, participants appeared to question the validity of national uptake data as a result of varied delivery models and data extraction methods, and the metrics requested by Public Health England.(Riley et al., 2018) |
| CMO 23 | Commissioners will be less inclined to utilise data (O) if they cannot operationalise them (M). This can occur in a context of not being able to transform data into ‘information’ (C) | Ackoff's theory of data-information-knowledge-wisdom (DIKW) | *But for us as commissioners even if we have the data, we need to know is that a good thing or a bad thing? Well I’ve no idea. It doesn’t mean anything. We have information about different services provided . . . but the information is fundamentally a garbage metric whichever way you look at it.*(Currie et al., 2018)  *So we get high-level data that says, you know, we’ve got X many cataracts happening, but we wouldn’t have the time to go into how is that different from everywhere else, and why is it different from everywhere else?*(Hollingworth et al., 2015) |
| CMO 24 | Having a data champion within the commissioning team support and promote the usage of data in commissioning decisions (C) can increase the usage of data (O) because the data champion can increase engagement and persuade people to use data (M) | Data champion. A data champion is a concept used to develop the CMO | First, there appears to be a link between those HAs with a ‘fully operational’ GIS and those that have a designated  person who is responsible for the maintenance of GIS throughout the organization. This might suggest that those organizations that have made the most progress, since the time of the previous survey, have been those that have invested in personnel dedicated to GIS, and have thus benefited from increased levels of commitment. However, there does not appear to be a link between those HAs that have fully operational GIS and whether GIS-based analysis is being supplied and administered from a central IT department. This suggests that, although having staff specifically providing a GIS service is likely to increase the sophistication of the resultant GIS analysis, it is not necessarily the case that such a service is provided by the IT service per se , and that GIS ‘champions’ who may work in other departments are likely to be just as influential, if not more so.(Smith et al., 2003)  The tool necessitated GP practice-level data and generated output on patients that were at higher risk of using health services. One superuser’s role was to embed the software tools into GP practices and encourage GP practice staff to use them. In transforming her learning about the software tool to GP practices, she selected aspects of the webinar learning that were germane to practice managers and administration staff and dejargonised the language.(Wye et al., 2015a) |
| CMO 25 | When commissioners lack the skills to analyse and interpret data (C) they cannot understand and draw insights from data for commissioning decisions (O) because of the knowledge gap (M) |  | Many of these data sources, skills and tools are already available within PCTs, but there is often a lack of capacity to make full use of the data in a manner that is timely and appropriate for commissioning decisions.(Wade et al., 2006)  *“I think we already have plenty of information to use...the skill is in interpreting it correctly and in making sure it matches the reality….”*(Holmes et al., 2009)  *We very rarely look at cost utility calculations, mostly because they’re not available, and they’re quite often very difﬁcult to do.(Williams and Bryan, 2007)* |
| CMO 26 | If commissioners want to persuade others about their commissioning proposals (C) they may (selectively) use data to support their proposals (O) due to a perception that they are a form of evidence that are ‘objective’ and can increase the legitimacy of proposals (M) | Political model of research utilisation (Weiss) | This leaves PCTs open to the accusation of using evidence and decision tools primarily as a legitimizing device rather than as a driver of decision outcomes.(Robinson et al., 2012)  Public health data were used to support and legitimise emerging commissioning plans, rather than drive them from the outset. Once a ﬁrm decision was made to move ahead with service redesign—as with the diabetes plan and early supported discharge service in Somerset (columns 3 and 4, table 3) and memory assessment service in Wirral (column 6, table 3)—progress seemed to speed up, and the service model, referral procedures and stafﬁng were established within months rather than years.(Shaw et al., 2013)  *If I’m being honest, there’s probably some retro fitting of that information to the things that the GPs want to do. So kind of regardless of all of that analysis, from the experience of the GPs in their consulting room the key issue is with the elderly and we need to be more integrated.*(Currie et al., 2018) |
| **Category: interpersonal relationships with and perceptions of external providers** | | | |
| CMO 27 | If commissioners perceive external support to be able to provide different or new skills (C) commissioners will be more inclined to use the data and outputs they produce (O) because they are perceived as novel and useful (M) | Theory of coproduction | In summary, external providers were brought in for their knowledge, skills and extra manpower.(Wye et al., 2015a)  In particular, the strength of the external teams lay in their ability to provide more in-depth analytical responses to complex questions from CCGs:  *So it’s the questions that are much more difficult to answer, so things like ‘what are the impacts of doing X on Y? What’s the likely demand on our services in the future? How has this service done compared to another service in another area?’ or ‘What is the evidence for this service to change?’. It’s kind of those more difficult questions which can’t necessarily be done with routine processing of data and reporting on routine data. We often have to link data and we have to provide a variety of different perspectives on a particular service or clinical area.*(Currie et al., 2018)  The ﬁrst of these areas, data analysis, was one where external support was perceived to have made an impact in all three PCTs. External organizations had brought in new analytical skills not previously present, for example, using risk stratiﬁcation techniques or novel approaches towards measuring patient experience. The information generated by these analyses was then being used to inform decision-making and to challenge the way providers deliver services to patients.(Naylor and Goodwin, 2011) |
| CMO 28 | When external providers understand the data needs of commissioners (C) and they can ‘co-produce’ the data (analysis) (C) commissioners are more likely to use their outputs (O), because they are relevant and useful (M) |  | Involving stakeholders throughout the process gave the infrastructure validity, codesigning a tool with end users ensured each element of the tool was necessary and enhanced the utility of the product for each clinical need. Regular communication with stakeholders throughout the design process and during the pilot phases allowed for continuous modification to the platform in use, making it more fit for purpose with each redeployment. Once more, involvement of stakeholders at each stage has encouraged uptake and repeated regular use of the tool. Stakeholders recognise that the product was developed to assist them in their day- to- day work and contains features they personally recommended. This codesign and feedback process will continue to focus the analysis and the development of additional features within the platform that fit the evolving needs of each practice, providing continuous support to optimise antibiotic prescribing.(Palin et al., 2020)  The need for adequate information was constantly cited as a problem:  *‘So we’d like to be able to think, to plan for savings and freed up resources, but it’s incredibly difﬁcult when we don’t have any budget statements from this year, or an end of year report from last year, and you know these are the frustrations that we are working with at the moment, we just don’t, we don’t have any information that we can manage our budgets on, you know if at the end of the year we are overspent, um, we only get told six months after the event, you know.. . has PBC failed or have the PCT failed? Because at what point can you start to take action to change that and make some remedial actions?’* (ID 34, practice manager)(Checkland et al., 2009) |
| CMO 29 | When commissioners can develop satisfactory relationships with the external providers of data (analysis) (C) commissioners are more likely to use data resulting from these relationships and collaborations (O) because the data produced are credible (M) |  | The commissioning manager in one case study explicitly  identiﬁed trust as the key factor in a productive commissioner– researcher relationship: *‘We need to trust each other – research people need to know that whatever they are trying to achieve, you will help them do that; the health authority have to trust them to let them do what you have agreed and to accept negative as well as positive results.’*(Harries et al., 1999)  There are some issues associated with this model (Petsoulas et al. 2014), and the arm’s length nature of CSUs does not necessarily make it easy for CCGs to build the required relationships in order to make best use of available data.(McDermott et al., 2015) |
| CMO 30 | In a context where there is a real or perceived divergence of interest (C) or information asymmetry between commissioners and external providers of data (analysis) (C) this may trigger feelings of mistrust among commissioners (M) which may make them less likely to use the data (O) | Principal-agent problem | *Quite often this sort of information is available from the actual drug companies themselves, isn’t it? I see that as a weakness with it. Because if a drug company is driving a product there may be the risk of bias.*(Williams and Bryan, 2007) |
